# Dipeptidase-1–knockout mice develop invasive tumors with features of microsatellite-unstable colorectal cancer

**DOI:** 10.1172/jci.insight.186938

**Published:** 2025-04-03

**Authors:** Sarah E. Glass, Matthew E. Bechard, Zheng Cao, Radhika Aramandla, Ping Zhao, Samuel T. Ellis, Emily H. Green, Elizabeth G. Fisher, Ryan T. Smith, Chelsie K. Sievers, Maria Johnson Irudayam, Frank Revetta, M. Kay Washington, Gregory D. Ayers, Cody N. Heiser, Alan J. Simmons, Yanwen Xu, Yu Wang, Annika Windon, Martha J. Shrubsole, Nicholas O. Markham, Qi Liu, Ken S. Lau, Robert J. Coffey

**Affiliations:** 1 Department of Cell and Developmental Biology, Vanderbilt University School of Medicine, Nashville, Tennessee, USA.; 2 Department of Medicine,; 3 Epithelial Biology Center,; 4 Vanderbilt Institute for Infection, Microbiology, and Immunology, and; 5 Department of Pathology, Microbiology, and Immunology, Vanderbilt University Medical Center, Nashville, Tennessee, USA.; 6 Cancer Biology Program, Vanderbilt University School of Medicine, Nashville, Tennessee, USA.; 7 Department of Biostatistics, Vanderbilt University Medical Center, Nashville, Tennessee, USA.; 8 Chemical and Physical Biology Program, Vanderbilt University, Nashville, Tennessee, USA.; 9 Center for Quantitative Sciences, Vanderbilt University Medical Center, Nashville, Tennessee, USA.; 10 Department of Pathology and Laboratory Medicine, Weill Cornell Medical Center, New York, New York, USA.; 11 Division of Epidemiology, Vanderbilt Epidemiology Center, Vanderbilt Ingram Cancer Center, Vanderbilt University Medical Center, Nashville, Tennessee, USA.; 12 Tennessee Valley Healthcare System, Department of Veterans Affairs, Nashville, Tennessee, USA.; 13 Center for Computational Systems Biology, Vanderbilt University, Nashville, Tennessee, USA.

**Keywords:** Cell biology, Oncology, Cancer

## Abstract

Dipeptidase-1 (DPEP1) is highly upregulated in colorectal cancer (CRC), with its enzymatic function linked to invasion and metastasis. More recently, DPEP1 was found to serve as a receptor for neutrophils when expressed by activated endothelial cells. It is unknown whether neutrophils bind to DPEP1-expressing CRC cells and whether this impacts features of CRC. Neutrophils have been shown to be tumor promoting in cancers including CRC, where they act to exclude CD8^+^ T cells. Herein, we show that neutrophils bind DPEP1-expressing CRC cells. In addition, *DPEP1* is preferentially expressed in microsatellite-stable (MSS) CRCs, in which there are a paucity of CD8^+^ T cells, whereas *DPEP1* is negatively correlated with microsatellite-unstable (MSI-H) CRCs, which are T cell rich and are more responsive to immunotherapy. Remarkably, carcinogen-treated *Dpep1*-null mice develop multiple, large, plaque-like, locally invasive adenocarcinomas and squamous cell cancers in the distal colon. These adenocarcinomas exhibit a marked reduction in neutrophils and an influx CD8^+^ T cells, along with reduced expression of mismatch repair proteins, consistent with features of MSI-H CRC. These results establish DPEP1’s importance in maintaining MSS CRC and its ability to shape the tumor microenvironment.

## Abstract 2

*DPEP1* is found to be a key gene in colorectal cancer tumor formation and maintaining microsatellite stability, impacting neutrophil and CD8^+ ^T cell recruitment.

## Introduction

Colorectal cancer (CRC), which accounts for almost 10% of cancer-related deaths in the world, has been well studied in the context of genetic changes that drive the normal epithelium toward a carcinoma (1). Seminal work by Eric Fearon and Bert Vogelstein outlined a stepwise accumulation of genetic events that leads to CRC (2). To identify candidate biomarkers and therapeutic targets, Vogelstein and colleagues curated their serial analysis of gene expression data for highly upregulated transcripts in both colorectal adenomas [Ad(s)] and cancers that encoded secreted or cell-surface proteins (3). They found that 1 of the 6 genes that met this criterion was *DPEP1*, or dipeptidase-1, which encodes a glycosylphosphatidylinositol-linked (GPI-linked) dipeptidase that is involved in extracellular leukotriene and glutathione metabolism (3–6). Upregulation of *DPEP1* in CRC has been confirmed by other investigators and has been linked to proliferation, drug resistance, invasion, and metastasis (7–14). We have shown that DPEP1 is increased in the plasma of a small subset of patients with CRC as compared with normal controls, hinting at its biomarker potential (15). In addition to its enzymatic activity, DPEP1 recently has been shown to act as a receptor for neutrophils when expressed on activated lung and liver mouse endothelial cells and assists in monocyte chemoattraction in the setting of inflammation (16, 17). *Dpep1*-null mice can survive a lethal dose of lipopolysaccharide due to the protein’s role in neutrophil recruitment, which can also affect the severity of acute kidney injury (17). To date, DPEP1 has only been studied for its enzymatic activity in the context of CRC, whereas its newly described role in neutrophil binding and its ability to shape the tumor microenvironment (TME) have not been examined in CRC (13, 18).

Immune infiltration is an important aspect concerning CRC outcomes, as higher levels of cytotoxic CD8^+^ T cells are associated with increased survival, whereas neutrophils can lead to CD8^+^ T cell exclusion and exhaustion and are associated with poor overall survival (19, 20). Immune cell infiltration has been correlated with deficiency in DNA mismatch repair proteins and microsatellite instability (MSI-H) (21). These errors in DNA proofreading lead to an increased neoantigen burden and immune cell infiltration within tumors and correlate with response to immune checkpoint inhibitors (ICIs) (21–25). However, the MSI-H subtype represents roughly 10%–15% of all CRCs, with the majority of CRCs having proficient DNA mismatch repair mechanisms and microsatellite stability (MSS), which is correlated with worse clinical outcomes (26, 27). Understanding how DPEP1 interacts with immune cells in CRC and how it relates to overall tumor phenotypes such as microsatellite status might expose a therapeutic vulnerability to overcome resistance of MSS CRC to ICI.

We have previously shown that *DPEP1* is part of a 4-gene immune exclusion (IEX) signature in MSS CRC that correlates with worse progression-free survival and CD8^+^ T cell exclusion from the tumor proper (28). The goal of this study was to determine the impact of DPEP1 on CRC tumorigenesis and the TME. Herein, we show that neutrophils bind to DPEP1-expressing CRC cells and that a lack of DPEP1 in carcinogen-induced colonic neoplasia results in adenocarcinomas (ACAs) that exhibit many of the features of MSI-H cancers, including a reduction in mismatch repair gene expression and an influx of CD8^+^ T cells into the tumor proper.

## Results

### Neutrophil binding to the CRC epithelium is DPEP1 dependent.

**Figure 1:**
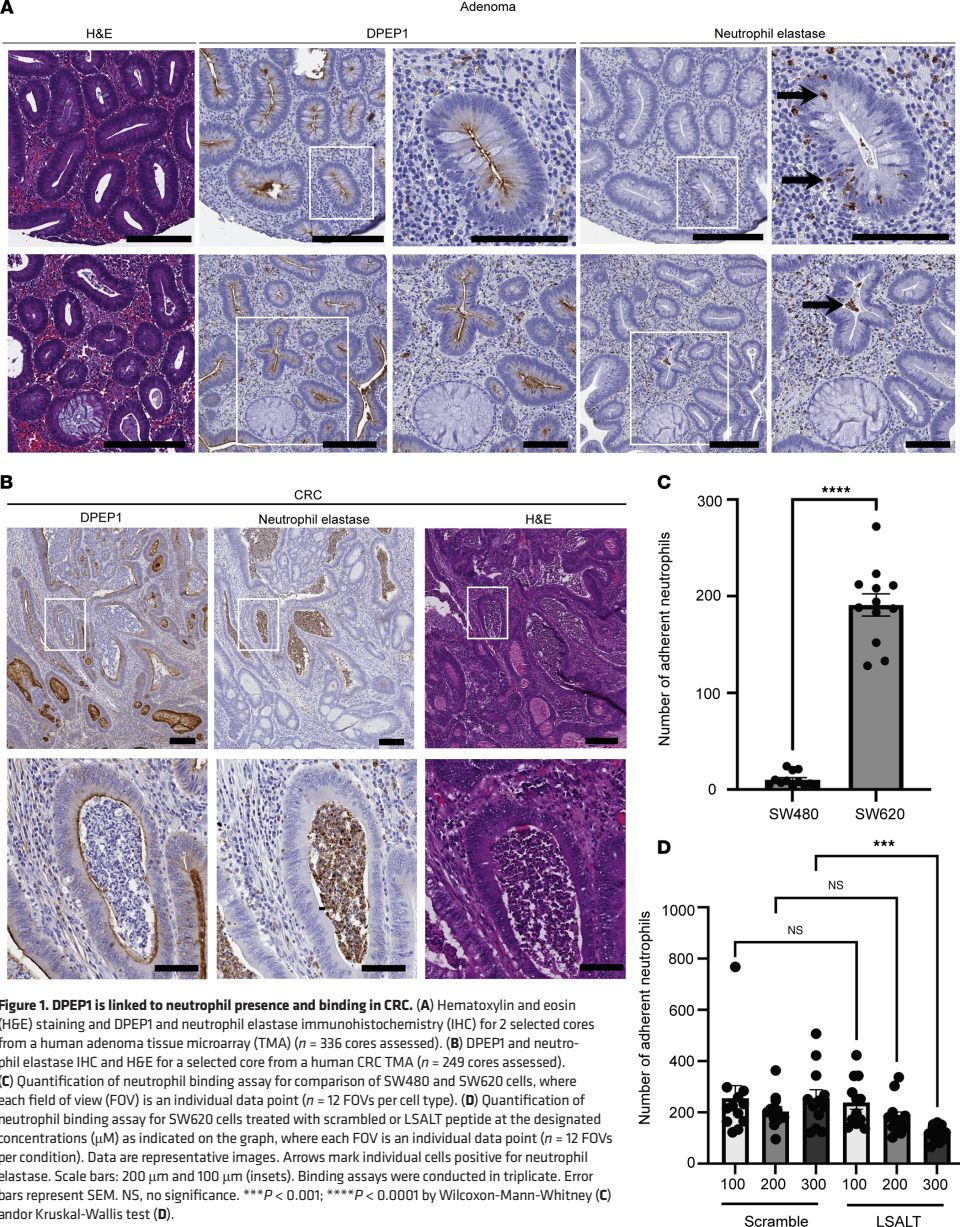
DPEP1 is linked to neutrophil presence and binding in CRC. (**A**) Hematoxylin and eosin (H&E) staining and DPEP1 and neutrophil elastase immunohistochemistry (IHC) for 2 selected cores from a human adenoma tissue microarray (TMA) (*n* = 336 cores assessed). (**B**) DPEP1 and neutrophil elastase IHC and H&E for a selected core from a human CRC TMA (*n* = 249 cores assessed). (**C**) Quantification of neutrophil binding assay for comparison of SW480 and SW620 cells, where each field of view (FOV) is an individual data point (*n* = 12 FOVs per cell type). (**D**) Quantification of neutrophil binding assay for SW620 cells treated with scrambled or LSALT peptide at the designated concentrations (μM) as indicated on the graph, where each FOV is an individual data point (*n* = 12 FOVs per condition). Data are representative images. Arrows mark individual cells positive for neutrophil elastase. Scale bars: 200 μm and 100 μm (insets). Binding assays were conducted in triplicate. Error bars represent SEM. NS, no significance. ***P < 0.001; ****P < 0.0001 by Wilcoxon-Mann-Whitney (**C**) andor Kruskal-Wallis test (**D**).

DPEP1 has recently been shown to be an endothelial adhesion receptor for neutrophils in the setting of inflammation, but whether DPEP1-expressing CRCs bind neutrophils has not been studied (16). *DPEP1* is upregulated in CRC by query of both the colon adenocarcinoma (COAD) and rectal adenocarcinoma (READ) datasets from The Cancer Genome Atlas (TCGA) in comparison with normal adjacent tissue (Supplemental Figure 1, A and B; supplemental material available online with this article; https://doi.org/10.1172/jci.insight.186938DS1) (3, 29). To determine the association between DPEP1 and neutrophils in colonic tumorigenesis, we performed immunohistochemical staining for DPEP1 and neutrophil elastase, a proteolytic enzyme secreted by activated neutrophils and monocytes during inflammation, on serial sections of colorectal Ad(s) and CRCs (30, 31). DPEP1 immunoreactivity was detected in 28% of Ad(s) from a tissue microarray (TMA) (Supplemental Figure 1, C and D). In many of the Ad(s), neutrophils were present in the stroma as well as traversing the epithelium (Figure 1A). DPEP1 immunoreactivity increased to 71% of CRCs upon analysis of a CRC TMA (Supplemental Figure 1, E and F), with its presence at the apical surface often associated with an accumulation of neutrophils in the lumen, so-called “dirty necrosis” (Figure 1B) (32–34). For Ad(s), there was a 70% concordance between DPEP1 and neutrophil elastase, which was increased to an 82% concordance for CRCs (Figure 1, A and B).

To test whether DPEP1-expressing CRC cells bind neutrophils, freshly isolated human neutrophils were plated on confluent cultures of SW620 cells, a metastatic CRC cell line reported to have high levels of DPEP1, and its primary tumor counterpart, SW480 cells, which express low levels of DPEP1 (Supplemental Figure 2A) (13, 35). Neutrophils bound to SW620 cells to a greater extent than SW480 cells (Figure 1C and Supplemental Figure 2B) (13). Treatment with LSALT, a 16–amino acid peptide reported to reduce binding of neutrophils to DPEP1, but not a scrambled peptide, led to a decrease in the number of neutrophils that bound to SW620 cells, suggesting that DPEP1 on SW620 cells binds neutrophils, but not the low-DPEP1-expressing SW480 cells (Figure 1D and Supplemental Figure 2, C–E) (16). Thus, neutrophils are able to bind CRC cells that express DPEP1.

### DPEP1 expression is regulated by Wnt activity and is associated with MSS status.

**Figure 2:**
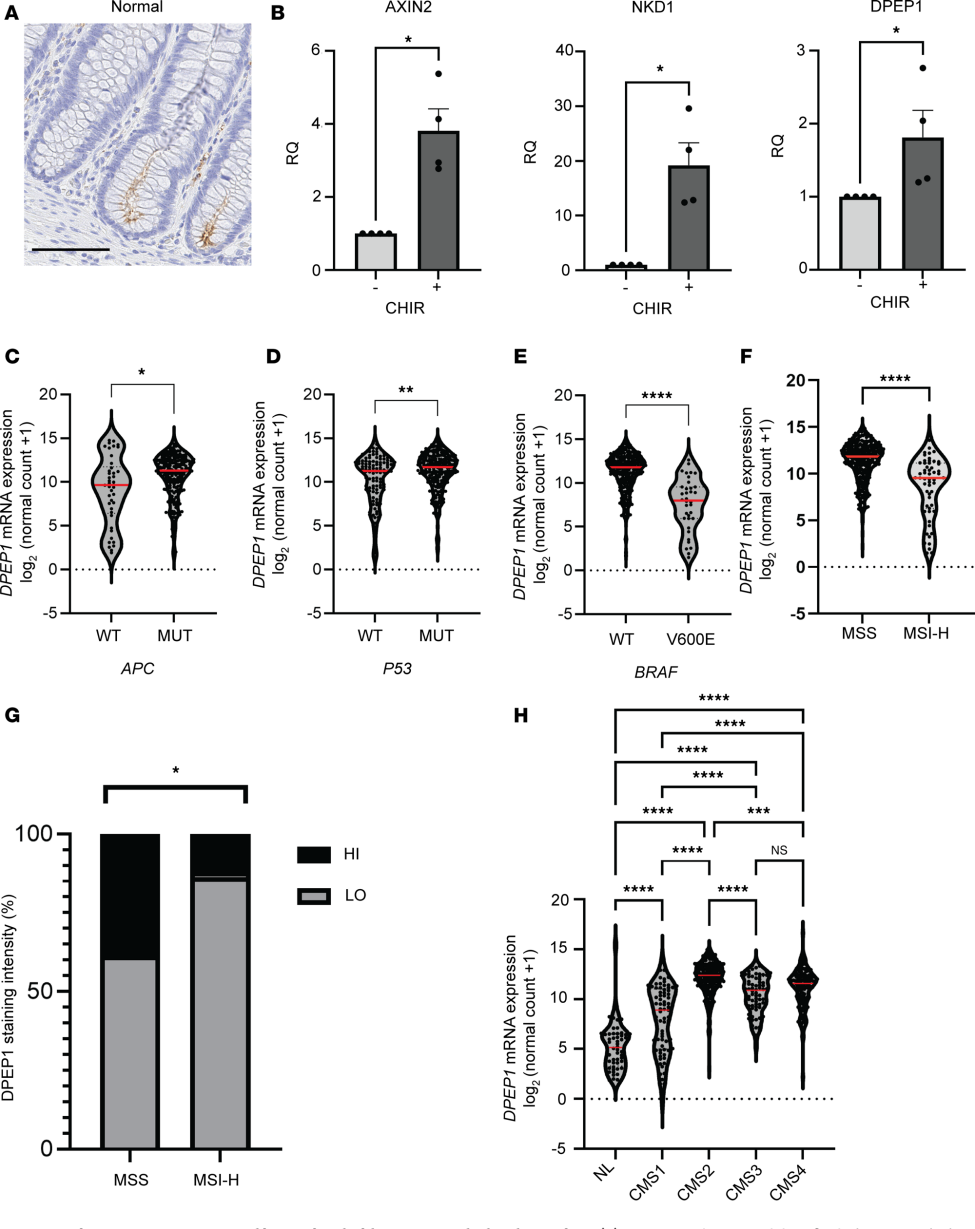
DPEP1 is a Wnt response gene and is associated with MSS CRC and related mutations. (**A**) Representative IHC staining of apical DPEP1 at the base of human normal colonic crypts. Scale bar: 100 μm. (**B**) Relative quantification for *AXIN2*, *NKD1*, and *DPEP1* mRNA levels in normal colonic organoids with or without CHIR99021 (CHIR) treatment (*n* = 4 biological replicates). Error bars represent SEM. (**C**–**F**) *DPEP1* mRNA expression of TCGA database COAD and READ cohorts as it relates to (**C**) *APC* (*n* = 274), (**D**) *P53* (*n* = 356), (**E**) *BRAF^V600E^* (*n* = 344) mutational status, and (**F**) MSI-H status (*n* = 375). (**G**) Percentage of CRC samples from TMAs based on DPEP1 staining intensity as delineated by MSI status (*n* = 105). (**H**) *DPEP1* mRNA expression of TCGA database COAD and READ cohorts as it relates to CRC CMS categories (*n* = 485). Median denoted in red. WT, wild-type; MUT, mutated; NS, no significance. *P < 0.05; **P < 0.01; ***P < 0.001; ****P < 0.0001 by Wilcoxon-Mann-Whitney, χ^2^ (**B**-**F**), chi squared (**G**), and Kruskal-Wallis test (**H**).

Upon closer examination of DPEP1 staining in the normal colon, we observed weak immunoreactivity on the apical surface of cells at the crypt base, where Wnt signaling is high (Figure 2A) (36). Since it is reported that *DPEP1* expression positively correlates with *CTNNB1* expression, which encodes the canonical Wnt signaling mediator β-catenin, we tested whether *DPEP1* expression was modulated by Wnt signaling (7). Treatment of normal human colonic organoids with CHIR99021, which activates Wnt signaling by GSK3 inhibition, led to increased expression of known Wnt-response genes *AXIN2* and *NKD1*, as well as *DPEP1* (Figure 2B) (37–39). Furthering DPEP1’s connection to Wnt signaling, our analysis of TCGA expression data shows that *DPEP1* is significantly upregulated in *APC*-mutant CRCs compared with CRCs lacking *APC* mutations (Figure 2C). *P53* mutations and pathogenic *NRAS* mutations also were associated with higher *DPEP1* levels, while *KRAS* and *SMAD4* mutations were not associated with *DPEP1* upregulation (Figure 2D and Supplemental Figure 3, A–C). Of interest, pathogenic mutations associated with MSI-H CRC (*BRAF^V600E^*, *PIK3CA*, and *PTEN*) were inversely correlated with *DPEP1* expression (Figure 2E and Supplemental Figure 3, D and E) (21, 40–42).

This prompted analysis of *DPEP1* expression in relation to MSI status using TCGA COAD and READ datasets. *DPEP1* expression was significantly higher in MSS CRC in comparison with MSI-H CRC (Figure 2F). This was confirmed at the protein level using the CRC TMAs (Figure 2G). Assessment of *DPEP1* expression across consensus molecular subtypes (CMS) classifications by using publicly available annotated CRC datasets from the Colorectal Cancer Subtyping Consortium (CRCSC) revealed that *DPEP1* was upregulated in all 4 CMSs in comparison with normal adjacent colon and rectal tissue (Figure 2H) (43). Of all the subtypes, *DPEP1* was most upregulated in CMS2, which is marked by Wnt signaling and is associated with MSS features (28, 43, 44). Altogether, these data support *DPEP1*’s upregulation due to Wnt signaling and its enrichment in MSS CRCs.

### Mice lacking DPEP1 during carcinogen-induced tumorigenesis have altered tumor burden, histology, and molecular features.

**Figure 3:**
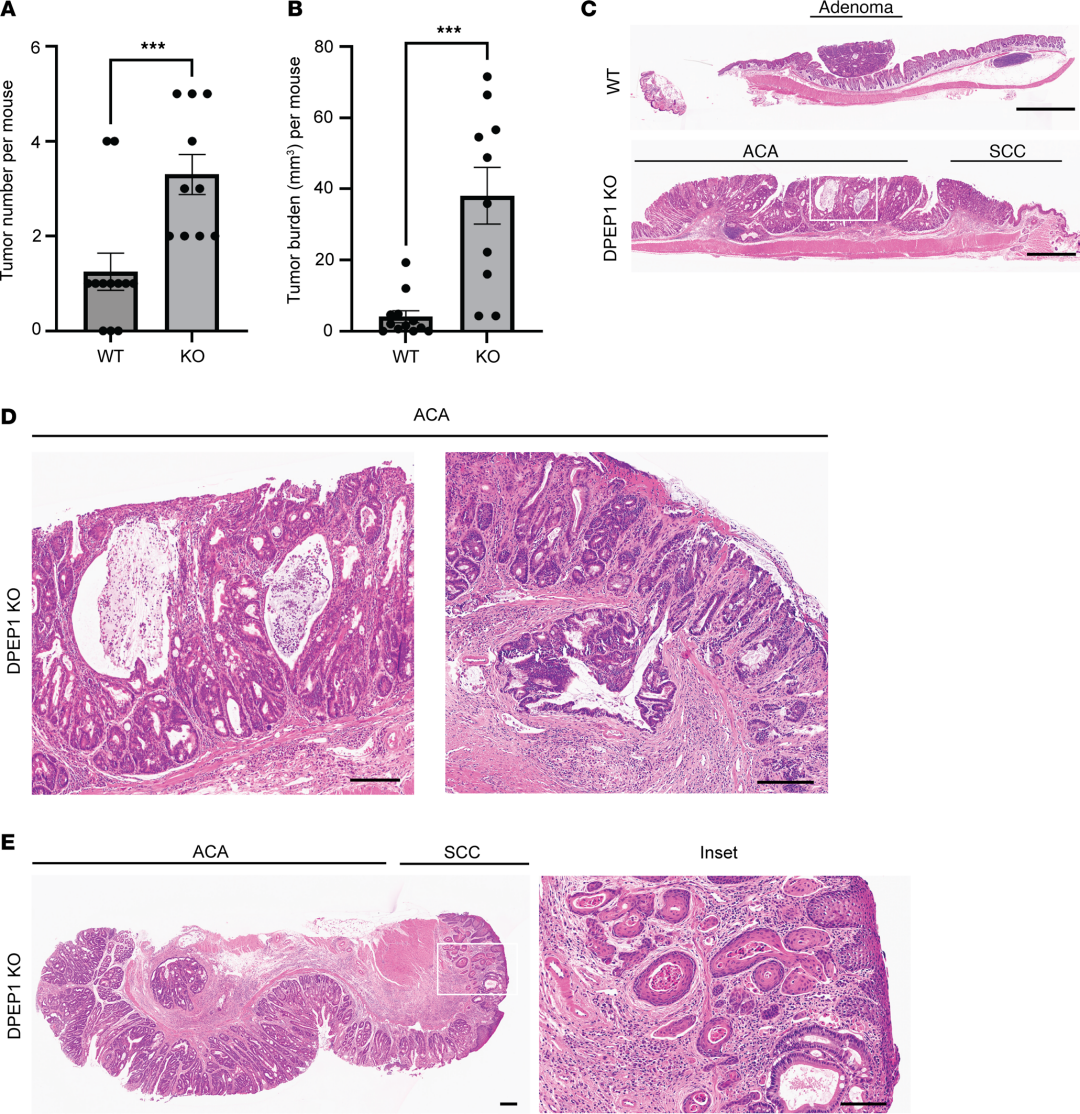
Mice lacking DPEP1 have an increased tumor burden and exhibit invasive adenocarcinoma and invasive squamous cell carcinoma. (**A**) Quantification of tumor number per mouse comparing WT (*n* = 12) and DPEP1-KO (*n* = 10) mice following a regimen of AOM/DSS. (**B**) Quantification of total tumor volume per mouse in WT (*n* = 12) and DPEP1-KO (*n* = 10) groups. (**C**) Representative H&E images of WT adenoma (Ad) and DPEP1-KO cancer. Scale bars: 1 mm. (**D** and **E**) H&E images of DPEP1-KO tumor shows 2 histological subtypes: (**D**) adenocarcinoma (ACA) with mucinous features as in the inset from **C** (left) and invasive features (right); and (**E**) squamous cell carcinoma (SCC), with related inset showing invasion. Scale bars: 200 μm. Error bars represent SEM. ***P < 0.001 by Wilcoxon-Mann-Whitney test.

As noted, we recently reported that *DPEP1* is a part of a 4-gene epithelial cell–intrinsic IEX signature in MSS CRC (28). This IEX signature was associated with an influx of neutrophils and a paucity of CD8^+^ T cells in the tumor proper and was correlated with worse overall and progression-free survival (28). It has also been shown that neutrophil infiltration early in the tumorigenic cascade can reduce CD8^+^ T cell infiltration into CRCs, at least in part, by secretion of metalloproteases that activate latent TGF-β (45). In addition, the release of neutrophil extracellular traps can create a shield for the tumor, allowing cancer cells to go unrecognized by cytotoxic CD8^+^ T cells (46). Based on our data connecting DPEP1 to neutrophils and MSS CRC, we utilized an azoxymethane/dextran sodium sulfate (AOM/DSS) model of colonic tumor formation in wild-type (WT) and *Dpep1^–/–^* (DPEP1-KO) C57BL/6 mice to understand the dynamics of DPEP1 in relation to histological features and immune cell recruitment (16). Validation of DPEP1 KO was confirmed by immunohistochemistry (IHC) and immunoblotting, with no overt histological, morphological, or phenotypic differences between WT and DPEP1-KO colons prior to AOM/DSS treatment (Supplemental Figure 4). After AOM/DSS treatment, DPEP1-KO mice had a markedly greater number of tumors than WT mice, as well as a greater tumor volume per mouse (Figure 3, A and B). Seventy percent of the DPEP1-KO mice, but none of the WT mice, had a prolapsed colon at time of sacrifice, likely reflecting the increased tumor burden. Histological examination of the colons revealed striking differences between WT and DPEP1-KO mice. In WT mice, we mostly observed single, small polyploid Ad(s) (Ads) (Figure 3C and Supplemental Figure 5A). In marked contrast, DPEP1-KO mice had sessile, plaque-like tumors with increased lamina propria and submucosal inflammation and invasion of the submucosa (Figure 3, C–E). Of note, 2 distinct subtypes of invasive tumor, both ACA and squamous cell carcinoma (SCC), were present in DPEP1-KO mice (Figure 3, C–E, and Table 1). The difference in tumor number and burden between WT and DPEP1-KO mice was still significant when only considering Ads and ACAs, highlighting that the presence of SCC was not the sole contributor in observed differences in tumor number and burden (Supplemental Figure 5, B and C). To investigate whether DPEP1 expression is modulated by colonic inflammation, mice underwent DSS treatment alone, without the use of AOM. DSS alone did not result in increased DPEP1 expression in the colon of non–tumor-bearing mice compared to untreated controls (Supplemental Figure 6, A–C).

Additionally, there were no differences in colonic inflammation or crypt damage between WT and DPEP1-KO mice only treated with DSS at days 5 and 19 after the start of DSS treatment (Supplemental Figure 6, D–H). In the AOM/DSS-treated mice that formed tumors, no differences in systemic inflammation were observed between WT and DPEP1-KO groups, as they had similar total white blood cell counts and absolute neutrophil counts (Supplemental Figure 6, J and K).

**Figure 4:**
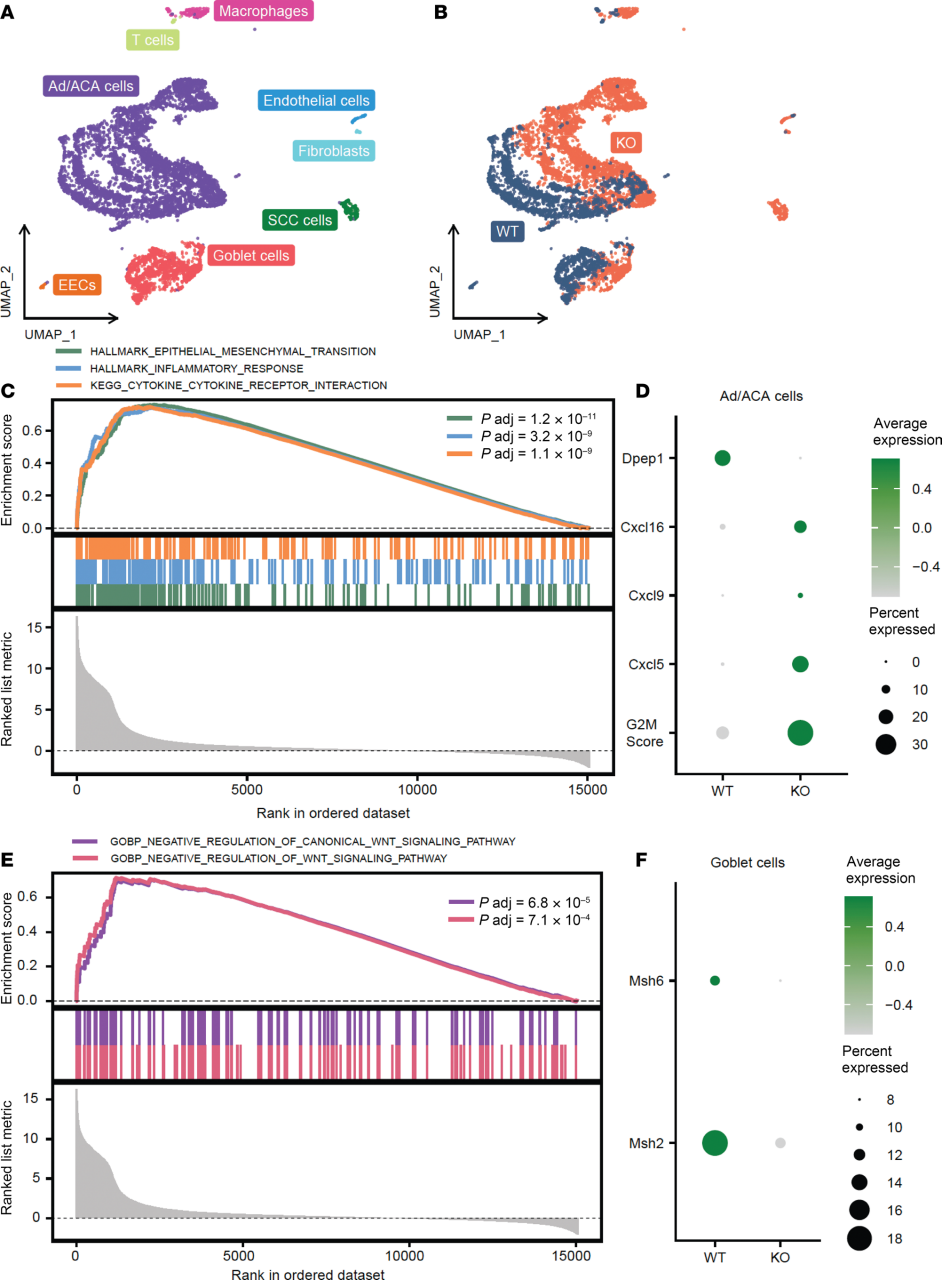
Mice lacking DPEP1 form tumors with molecular features distinct from MSS. (**A**) UMAP representation of the major cell types isolated from WT and DPEP1-KO tumor tissue. (**B**) UMAP plot showing the WT and DPEP1-KO groups. (**C**) FCS-GSEA plots showing example significantly enriched signaling pathways in DPEP1-KO Ad/ACA cells in comparison with WT. (**D**) Dot plot of *Dpep1*, select chemokine genes, and G2M score in WT and DPEP1-KO groups in Ad/ACA cells (*Dpep1* *P*_adj_ = 1.2 × 10^–139^; *Cxcl16* *P*_adj_ = 7.6 × 10^–23^; *Cxcl9* *P*_adj_ = 5.5 × 10^–13^; *Cxcl5* *P*_adj_ = 8.6 × 10^–77^; G2M score *P*_adj_ = 1.6 × 10^–61^). (**E**) FCS-GSEA plots showing the significantly enriched pathways in DPEP1-KO Ad/ACA cells, which are negatively regulated WNT signaling pathways. (**F**) Dot plot of DNA repair genes in WT and DPEP1-KO groups in goblet cells (*Msh2* *P* = 1.2 × 10^–3^; *Msh6* *P* = 6.3 × 10^–2^). *P*_adj_, adjusted *P* value; EECs, enteroendocrine cells.

To gain insight into the molecular nature of these colonic neoplasms, we performed single-cell RNA sequencing (scRNA-seq). The analysis revealed distinct populations of cells in uniform manifold approximation and projection (UMAP) space, with cell type annotation informed by distinct markers consistent with each cell type (Figure 4A and Supplemental Figure 7A). SCC cells were only found in DPEP1-KO mice (Figure 4, A and B). The top 25 differentially expressed genes between ACA cells and SCC cells from DPEP1-KO tumors are depicted in Supplemental Figure 7B. Functional class scoring gene set enrichment analysis (FCS-GSEA) of the Ad/ACA cell population revealed that the Hallmark Epithelial-Mesenchymal Transition, Hallmark Inflammatory Response, and Kyoto Encyclopedia of Genes and Genomes (KEGG) Cytokine–Cytokine Receptor Interaction pathways were significantly enriched in DPEP1-KO cells in comparison with WT (Figure 4C), consistent with the invasive nature of the DPEP1-KO ACAs. Analysis of individual genes in the Ad/ACA cell population revealed that *Dpep1* was enriched in the WT condition, as expected (Figure 4D). We found that expression of cytokines associated with T cell and neutrophil infiltration was increased in DPEP1-KO Ad/ACA cells (Figure 4D) (47–49). The G2M score also was enriched in DPEP1-KO Ad/ACA cells in comparison with the WT counterpart, in agreement with the larger tumor size in DPEP1-KO mice (Figure 4D). We also discovered that DPEP1-KO Ad/ACA cells were enriched for the FCS-GSEA categories related to negative regulation of Wnt signaling, which was confirmed by association of Wnt negative and positive regulators in DPEP1-KO Ad/ACA cells in comparison with WT (Figure 4E and Supplemental Figure 7, C and D). In line with DPEP1’s association with MSS CRC, we found that DPEP1-KO goblet cells had significantly less expression of two DNA mismatch repair markers, Msh2 and Msh6, in comparison to WT goblet cells (Figure 4F) (50). While DPEP1 is reported as a receptor for neutrophils in the liver and lung endothelium, it is important to note that we did not see significant expression of *Dpep1* in the endothelial cells isolated from the tumors of WT mice (Supplemental Figure 7E).

### Mice lacking DPEP1 form ACAs with MSI-like characteristics, including altered DNA repair marker expression and an influx of CD8^+^ T cells.

**Figure 5:**
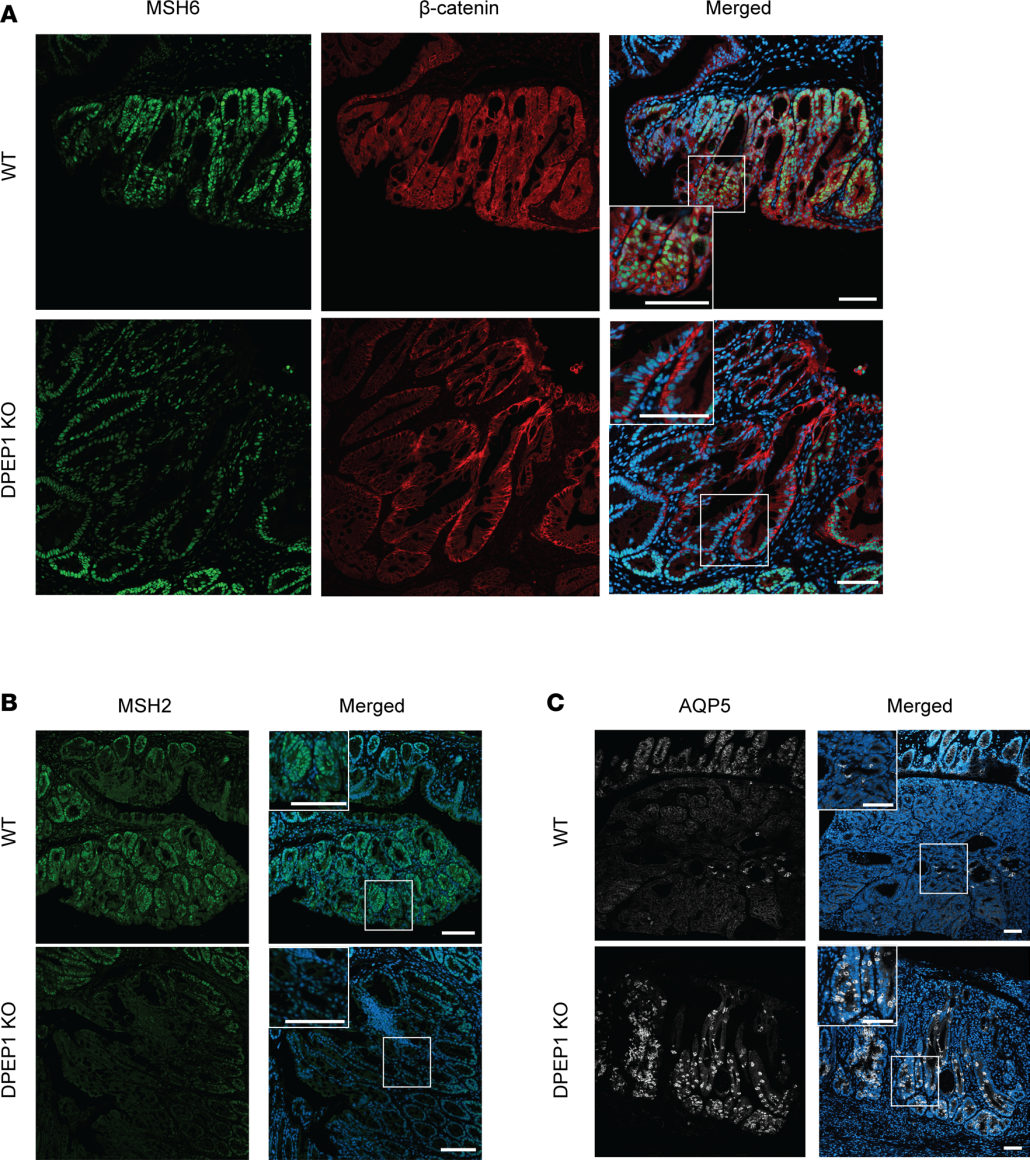
DPEP1-KO mice display features of microsatellite instability in epithelial cells. Representative immunofluorescence images for WT Ads and DPEP1-KO ACAs stained for (**A**) MSH6 and β-catenin or (**B**) MSH2 or (**C**) AQP5 and merged with DAPI staining. Scale bars: 100 μm. Representative images are a result of staining tumors from 2 cohorts described in the Methods, where experiments were done in triplicate from WT (*n* = 12) and DPEP1-KO (*n* = 10) tumors.

**Figure 6:**
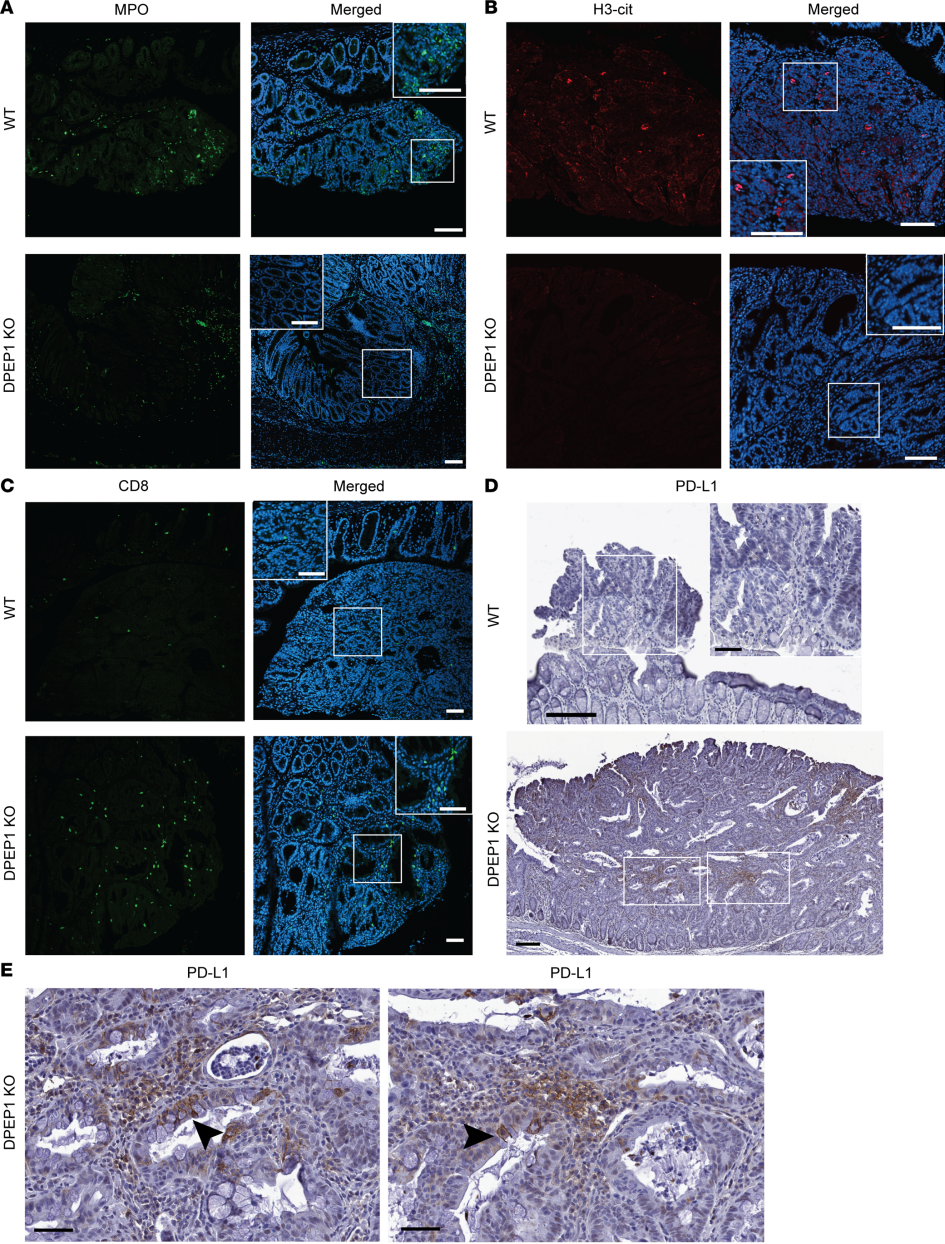
DPEP1-KO mice present with immune-related features of microsatellite instability. Representative immunofluorescence images for WT Ads and DPEP1-KO ACAs stained for (**A**) MPO, (**B**) H3-cit, and (**C**) CD8 merged with DAPI staining. Scale bars: 100 μm. (**D** and **E**) Representative PD-L1 IHC staining for WT Ads and DPEP1-KO ACAs. Scale bars: 150 μm (**D**) and 50 μm (**E** and insets in **D**). Representative images are a result of staining tumors from 2 cohorts described in the Methods, where experiments were done in triplicate from WT (*n* = 12) and DPEP1-KO (*n* = 10) tumors.

To further investigate the role of DPEP1 in altering the tumor phenotype, we examined a number of relevant proteins by immunofluorescence. WT Ads showed enhanced expression of DPEP1, whereas, as expected, DPEP1-KO ACAs were devoid of DPEP1 expression (Supplemental Figure 8A). In particular focal regions of the DPEP1-KO ACAs, expression of the DNA repair proteins MLH1, MSH2, and MSH6 was much reduced in comparison with Ads in WT mice (Figure 5, A and B, Supplemental Figure 8B), consistent with scRNA-seq results (51). In regions with reduced MSH6 staining in the DPEP1-KO condition, we noted a prominent basal localization of β-catenin, consistent with β-catenin acting as a component of adherens junctions, as opposed to diffuse β-catenin staining in the WT condition that reflects activation of canonical Wnt signaling (Figure 5A) (52). Furthermore, serial sections of DPEP1-KO colonic ACAs showed patchy losses with similar distributions of MSH2 and MSH6 staining in comparison with WT DPEP1 Ads (Figure 5A and Supplemental Figure 8C). DPEP1-KO ACAs also had increased aquaporin-5 (AQP5) expression in comparison with the WT condition, which we and others have reported to be consistent with MSI-H tumors and their precursor sessile serrated lesions (Figure 5C) (28, 53, 54). Since DPEP1 acts as a receptor for neutrophils, we next examined whether the absence of DPEP1 would affect the neutrophil census. In the WT condition, we observed staining for myeloperoxidase (MPO), citrullinated histone H3 (H3-cit), and neutrophil elastase, markers of activated neutrophils, whereas these were all much reduced in DPEP1 KO (Figure 6, A and B, and Supplemental Figure 9) (20, 28, 55). Specifically, MPO staining marked neutrophils that were intercalated among epithelial cells in the WT condition but were confined to the stroma in DPEP1-KO ACAs (Supplemental Figure 9A). Consistent with neutrophils reportedly excluding CD8^+^ T cells, we saw a marked increase in CD8^+^ T cells within the ACAs of DPEP1-KO mice compared with WT, further supporting our suspicion that a lack of DPEP1 confers features of MSI-H CRC (Figure 6C). An additional feature of MSI-H CRC tumors is increased PD-L1 staining, which we observed to be higher in DPEP1-KO ACAs in comparison with WT (Figure 6, D and E) (54, 56). Taken together, these data provide evidence that DPEP1 has a causal role in immune exclusion, which we define as a paucity of CD8^+^ T cells in the tumor proper, and its expression appears to be critical for the maintenance of an MSS phenotype (28).

## Discussion

Eleven years after establishing the Vogelgram, Vogelstein and colleagues identified *DPEP1* as 1 of 6 genes encoding a secreted or cell-surface protein that was upregulated 20-fold or greater in colorectal Ad(s) and CRCs (3). We now show that DPEP1 immunoreactivity steadily increases during CRC progression, with DPEP1 detected in 28% of Ad(s) and 71% of CRCs. These results are in alignment with a study positing DPEP1 as a urinary biomarker for both Ad(s) and CRCs (57). In the normal colon, we observed occasional weak staining for this GPI-linked protein at the apical surface of epithelial cells at the crypt base, where Wnt-driven stem cells reside (36). Furthermore, activation of canonical Wnt signaling in normal human colonoids increased expression of *DPEP1*, as well as known Wnt response genes, *AXIN2* and *NKD1* (38, 39). In addition, we found that DPEP1-KO ACAs were associated with negative regulators of Wnt signaling. The increased β-catenin expression in DPEP1-KO Ad/ACA cells in comparison with WT may reflect the enhanced β-catenin staining we consistently observed at the basal membrane in the DPEP1-KO setting. This pattern of β-catenin staining has recently been linked to an actin-based basal biomechanical process that coordinates epithelial tissue stability and organization (58). Beyond the finding that *DPEP1* expression is positively correlated with *CTNNB1* expression, there are no reports looking at the regulation of *DPEP1* by Wnt signaling (7). We propose that there may be a positive feedback loop where DPEP1 regulates Wnt signaling, as there is a loss of active Wnt signaling as evidenced by the apparent accumulation of β-catenin at the basolateral membrane in the DPEP1-KO mice after a regimen of AOM/DSS. MSS CRC, which makes up the majority of CRCs, is known to commonly have APC loss of function and heightened canonical Wnt signaling (2, 25, 53). Thus, it is not surprising that DPEP1 is preferentially increased in MSS CRCs in comparison with MSI-H CRCs, along with a number of mutations that are associated with MSS CRCs such as *APC*, *TP53*, and *NRAS* (2, 50, 59). Furthermore, we found that increased *DPEP1* expression is inversely correlated with MSI-H CRCs and associated mutations, such as *BRAF^V600E^*, *PIK3CA*, and *PTEN* (41, 42, 50, 54).

We also investigated DPEP1’s functional role in interacting with neutrophils, which has been reported only in an endothelial cell context (16). We found that DPEP1 on CRC cells binds neutrophils within 30 minutes. As there is evidence that Wnt signaling impacts other molecules important for neutrophil adhesion, such as ICAM-1 and VCAM-1, future work might involve determining the individual contributions of DPEP1 and other neutrophil-binding molecules in neutrophil recruitment to the tumor and how Wnt signaling might regulate this interplay (60).

The most unexpected finding in this study was the histological features of the tumors that developed following AOM/DSS treatment of DPEP1-KO mice. In contrast with the solitary, pedunculated tumors in WT mice that exhibited adenomatous features histologically, we observed multiple, large, sessile, plaque-like tumors in the very distal colon of DPEP1-KO mice. Equally surprising was the co-occurrence of locally invasive mucinous ACAs and separate invasive SCCs. After AOM/DSS treatment, DPEP1-KO mice exhibited features of human MSI-H CRCs, such as reduced expression of DNA repair proteins, altered Wnt signaling, and an infiltration of CD8^+^ T cells (27, 28, 53, 61). We speculate that DPEP1 expression restricts tumor development toward canonical Wnt signaling–dependent, stem cell–driven tumor formation akin to CMS2, whereas its absence leads to the development of a different CRC subtype that is reminiscent of metaplastic sessile serrated lesions developing into a plaque-like MSI-H CRC, akin to CMS1 (28, 44, 53). Use of the *Lrig1-CreER^T2/+^;Apc^fl/+^* mouse model, which results in inducible Wnt-driven distal colonic tumor formation, could complement the present study to determine the role of DPEP1 in a tumorigenic cascade that is biased toward an MSS fate, in contrast with the AOM/DSS model which is more inflammatory in nature (62, 63).

There is evidence that alteration of Wnt signaling can impact DNA mismatch repair gene expression, leading to the promotion of an MSI-H state in CRC that improves responsiveness to ICI (64). We show that mice lacking DPEP1 display colonic neoplasia with areas of reduced mismatch repair protein expression corresponding to areas of the ACA with a basal membrane β-catenin staining pattern in comparison with a cytosolic and nuclear staining pattern in the WT condition. This reduction of Wnt signaling in a DPEP1-KO context could be an early contributing factor to the development of MSI-H-like features. Also, we noticed that areas with reduced expression of MSH2 exhibited reduced expression of its binding partner, MSH6, suggesting that lack of one DNA repair marker could lead to destabilization of the other (65). Our scRNA-seq results demonstrated that the reduced expression of the mismatch repair genes *Msh2* and *Ms*h6 was significant in goblet cells isolated from ACAs of the DPEP1-KO mice in comparison with Ads of the WT mice. This is consistent with our previous findings that sessile serrated lesions, a precursor to MSI-H CRC, arise from gastric metaplasia, not Wnt signaling, with the main contributors to a non–stem cell–driven CRC being goblet and enteroendocrine cells, differentiated cells that reside above the colonic crypt base (28, 53). Our data also highlight the potential role of goblet cells in MSI-H tumorigenesis, as their association with loss of mismatch repair proteins is not well appreciated in the literature. Overall, a deeper understanding of the make-up of these tumors could lead to the identification of signaling pathways important for DPEP1-dependent tumor formation that allows for a distinctly different tumor milieu in terms of immune composition.

In DPEP1-KO mice, we discovered that some of the traditional signs of immune exclusion have been reversed and that the formation of tumors, although invasive, have an influx of CD8^+^ T cells, which is a favorable predictive factor for response to ICI (23, 66). Although it is perhaps surprising that DPEP1-KO mice had a greater tumor burden, this is reflective of patients whose MSI-H CRCs tend to be larger at presentation than their MSS counterparts (67). It is possible that these locally invasive ACAs may be more responsive to ICI due to infiltration of CD8^+^ T cells and presence of PD-L1.

A number of studies have reported a role for DPEP1 in CRC, showing its upregulation in tumor tissue and the connections between its well-established enzymatic activity and tumorigenesis (7–14, 68). While DPEP1’s nonenzymatic neutrophil-binding activity has been described in relation to sepsis and acute kidney injury with the LSALT peptide being used for COVID-19–related clinical trials, there have been no previous reports connecting its neutrophil-binding activity to cancer (16, 17, 69). Micromolar amounts of LSALT peptide were necessary to achieve a significant effect on neutrophils binding to CRC cells, consistent with the original study reporting its use, highlighting the need for the development of more potent inhibitors (16). Nevertheless, we have created a foundation for nonenzymatic functional studies of DPEP1 by showing that DPEP1-dependent neutrophil binding occurs in CRC as well as reduced neutrophil infiltration into the tumors of DPEP1-KO mice.

Neutrophils can be both pro- and antitumorigenic based on the context (70). The neutrophil-to-lymphocyte ratio (NLR) has been shown to impact disease-free and overall survival as well as responsiveness to ICI, with a high NLR leading to worse outcomes (71, 72). We find that DPEP1 expression is linked to the presence of neutrophils in the stroma and accumulation in the luminal space, which might allow for immune exclusion of other cell subsets such as cytotoxic CD8^+^ T cells. Interestingly, the absence of “dirty necrosis” or neutrophil accumulation in the lumen has been associated with an MSI-H CRC phenotype (32–34). Even though DPEP1-KO Ad/ACA cells expressed *Cxcl5*, a potent chemoattract for neutrophils, we did not see a concomitant influx of neutrophils, highlighting that other factors, such as DPEP1, impact neutrophil recruitment (49). We also show that neutrophil binding to CRC cells has a DPEP1-dependent component by addition of LSALT peptide. We furthered this finding by inducing tumor formation in DPEP1-KO mice to show a reduction in cells with markers for neutrophil activation and an influx of CD8^+^ T cells, highlighting DPEP1’s causal role in immune exclusion rather than just being a gene that is part of an IEX signature (28). Other cell subsets beyond neutrophils and T cells might be modulated during tumorigenesis since DPEP1 has also been shown to bind other monocytic populations (16, 17). Further analysis using complementary methods to the ones used here could reveal the role of DPEP1 in shaping additional components of the TME.

Although we cannot exclude the role of DPEP1’s enzymatic activity in the phenotypes seen with DPEP1-KO mice, the literature supports DPEP1’s interaction with neutrophils impacting CD8^+^ T cell exclusion (45). Along with the influx of CD8^+^ T cells in the DPEP1-KO setting, the Ad/ACA cells also expressed the chemokines *Cxcl16* and *Cxcl9*, which are important for T cell migration (47, 48). In particular, CXCL16 is associated with a good prognosis in CRC and T cell infiltration (47). Future studies that involve assessing the T cell phenotypes in these DPEP1-KO mice as well as tumor responsiveness to ICI could lead to the development of combination treatments for reversing an immune exclusion phenotype, as we found an upregulation of PD-L1 in the KO setting (24, 73, 74). The LSALT peptide has been used in phase II clinical trials for COVID-19 patients in an attempt to prevent acute kidney injury and acute respiratory distress syndrome, where it has been shown to be safe and well tolerated (69). Further studies assessing the impact of LSALT treatment on neutrophil recruitment and potentially concomitant CD8^+^ T cell exclusion in CRC or in other cancers could lead to a viable therapeutic to be combined with ICI. With this study as a base, future work can build on the clinical relevance of our basic biological findings that connect DPEP1 to immunomodulation in a CRC context. Overall, this study clarifies DPEP1’s association with Wnt-driven MSS CRC and opens the door for exploring DPEP1’s immunomodulatory roles in cancer that could be widely translatable, with the goal of making MSS tumors responsive to ICI.

## Methods

### Sex as a biological variable.

For this study, sex as a biological variable was accounted for by ensuring that equal numbers of male and female mice were used for AOM/DSS experiments. Male mice exhibited more tumors and larger tumors than female mice, as has been previously reported (75).

### Cell lines and culture.

SW480 and SW620 cell lines were obtained from the American Type Culture Collection. These cells were cultured in DMEM supplemented with 10% bovine growth serum, 1% glutamine, 1% non-essential amino acids, and 1% penicillin-streptomycin at 37°C in a 5% CO_2_ humidified incubator.

### Organoid culture and CHIR99021 treatment.

Human normal colonic organoids were spilt into a single-cell suspension using TrypLE (Gibco) for 3 minutes at 37°C with manual disruption and plated at 700 cells/μL in 30 μL Matrigel (Corning) domes. Organoids were cultured for 7–10 days in Human IntestiCult (STEMCELL Technologies) media at 37°C, 5% CO_2_. For experimental wells, the GSK3β small molecule inhibitor, CHIR99021 (Tocris, 4423), diluted in DMSO (Thermo Fisher Scientific) was added to the medium at a final concentration of 10 μM for 16 hours. Organoids were washed for 15 minutes in 1× PBS.

### Animal studies.

Male and female WT C57BL/6 (The Jackson Laboratory) and *Dpep1^−/−^* (DPEP1-KO) mice (gift from Donna L. Senger, McGill University, Montreal, Canada) were used for these studies in a cohort of 8 mice per group (10 weeks of age) and a second repeat experiment of 4 mice per group (22 weeks of age). A third repeat experiment with 7 mice per group (11–15 weeks of age) was used that were also heterozygous or homozygous carriers of the previously described neutrophil reporter construct *Ly6g* (*Cre-tdTomato*) (76). The DPEP1-KO mouse line used carries the 1–base pair deletion that is designated “C” in the original paper (16). Mice were injected intraperitoneally (i.p.) with 10 mg/kg of AOM (Sigma-Aldrich) in PBS once a week for 2 weeks. One week after the second AOM injection, mice were given ad lib access via water bottle to 2% colitis-grade DSS for 5 days. After approximately 3 months of growth, tumor number was counted, and tumor volume was measured with calipers and calculated as follows: volume = (*w* × *h* × *d*)/2, where *w* is the shortest diameter, *h* is the longest diameter, and *d* is the depth. Three DPEP1-KO mice died before 3 months and therefore were removed from the analysis. A control cohort with WT and DPEP1-KO mice (15–32 weeks of age) underwent treatment for 5 days with 2% DSS–containing water, without prior AOM injections. These were sacrificed on the final day of DSS treatment or 14 days following completion of DSS treatment. For assessing DSS damage, inflammation, percentage involved in inflammation, depth of inflammation, crypt damage, and percentage involved in crypt damage were scored by an expert gastrointestinal pathologist.

### Data collection for mutation, mRNA, and protein expression correlations.

The University of California Santa Cruz Xena browser (https://xenabrowser.net/) was used to download TCGA COAD and READ datasets for assessing mRNA expression of *DPEP1* as well as the CRCSC (https://doi.org/10.7303/syn2623706) for downloading CMS information for patients in TCGA COAD and READ datasets (77). TCGA data were downloaded as log_2_-transformed, normalized counts. The mutation and mRNA results are based on data generated by TCGA Network (https://www.cancer.gov/tcga).

### RNA isolation and qRT-PCR assay.

Total RNA was extracted using 1 mL of TRIzol reagent (Invitrogen) for 10 minutes with manual disruption using a sterile P1000 pipette tip. RNA clean-up was performed using the Direct-zol RNA MiniPrep Plus kit (Zymo Research) according to the manufacturer’s protocol. cDNA was generated using the Verso cDNA synthesis kit (Thermo Fisher Scientific). Real-time quantitative reverse transcription PCR (qRT-PCR) was performed using TaqMan Fast advanced master mix and genetic probes (Thermo Fisher Scientific) in a QuantStudio 6 Flex PCR machine (Applied Biosystems) per the manufacturers’ recommendations. Relative units (RUs) were quantified comparing organoids with and without CHIR99021 utilizing β-actin (*Actb*) as an endogenous control. RUs were determined by subtracting the cycle threshold (Ct) of the gene of interest from the Ct of β-actin, yielding the ΔCt. The ΔΔCt was calculated by subtracting the ΔCt of the untreated sample from the ΔCt of the experimental sample for each gene. RUs were then equal to 2^–ΔΔCt^. The following primer probe sets were used from Thermo Fisher Scientific: *ACTB* (Hs01060665_g1), *NKD1* (Hs01548773_m1), *AXIN2* (Hs00610344_m1), and *DPEP1* (Hs01116752_m1).

### IHC.

Human tissue samples were obtained under IRB-approved protocols from the umbrella spore IRB, Tennessee Colorectal Polyp Study (TCPS), and collaborators at John Hopkins University. The polyp tissue used to construct the adenoma TMA was obtained from participants in the TCPS, who were of 40–75 years of age and did not have a genetic CRC syndrome or prior history of inflammatory bowel disease (IBD). The CRC tissue used to construct the CRC TMA was obtained from CRC patients who had no history of IBD, with the constructed TMA representing 173 patients. Staining was performed as previously described with antigen retrieval performed using pH 6.0 citrate buffer (15). The primary antibodies used were anti-DPEP1 (Sigma-Aldrich, HPA012783 [human specific] and Cell Signaling Technology, 87223 [human specific] and 84292 [mouse specific]); anti–neutrophil elastase (Abcam, ab68672); and anti–PD-L1 (Cell Signaling Technology, 64988). Staining with Gill 2 hematoxylin (Richard-Allan Scientific, 72504) and eosin (Sigma-Aldrich, HT110316) (H&E) was also performed. Scoring was performed by an expert gastrointestinal pathologist on a scale from 0 to 3. For these values, 0 and 1 were considered low expression, while 2 and 3 were considered high expression. For mouse studies, tumor tissues were formalin-fixed and paraffin-embedded (FFPE) by the Vanderbilt University Medical Center (VUMC) Translational Pathology Shared Resource (TPSR) and H&E staining was performed on resulting slides cut from FFPE blocks as described above.

### Immunofluorescence.

Tissue sections of 5 μm thickness were first blocked with 5% normal donkey serum with 3% BSA in PBS at room temperature for 1 hour. After blocking, slides were incubated with primary antibodies against MSH6 (Abcam, ab92471), β-catenin (custom antibody produced in collaboration with the Vanderbilt Antibody Core), MSH2 (Abcam, ab212188), AQP5 (Abcam, ab215225), neutrophil elastase (Abcam, ab68672), histone H3 (citrulline R2 + R8 + R17) (Abcam, ab281584), MPO (Abcam, ab208670) CD8 (Cell Signaling Technology, 98941S), DPEP1 (Cell Signaling Technology, 84292 [mouse specific]), and MLH1 (Abcam, ab92312) overnight at 4°C. The next day, slides were washed and then incubated for 1–2 hours at room temperature with the appropriate fluorophore-conjugated antibody (conjugated to Alexa Fluor 488, 568, or 647; Thermo Fisher Scientific), and then washed again and mounted with DAPI-containing mounting media. Unless otherwise noted, images were acquired using a Nikon A1R laser confocal microscope.

### Immunoblotting.

Mouse tissues were lysed in CellLytic Mt Mammalian Tissue lysis/Extraction Reagent (Sigma-Aldrich) with a protease inhibitor tablet and a PhosSTOP tablet (Roche). Lysates were homogenized using 1.0 mm Zirconia beads (BioSpec Products) and a Mini BeadBeater-8 (BioSpec Products) for 1 minute according to the manufacturer’s protocol. Resulting lysates were then centrifuged at 17000*g* for 12 minutes after a 20-minute incubation at 4°C. For SW480 and SW620, the cells were cultured in 10 cm dishes and washed twice with ice-cold PBS and harvested in lysis buffer (25 mM Tris-HCl, pH 8.0, 0.5% NP-40, 50 mM NaCl, 0.5% sodium deoxycholate, 0.02% NaN_3_) with a protease inhibitor tablet and a PhosSTOP tablet (Roche). Lysates were pelleted at 13,300 rpm for 10 minutes, sonicated for 20 seconds, and then spun at 13,300 rpm for 10 minutes before isolation of the supernatant and addition of SDS sample buffer. Equal amounts (μg) of loaded sample were resolved in a 10% SDS-PAGE gel under reducing conditions, before transfer to a nitrocellulose membrane (Bio-Rad, 1704158) using a Trans-Blot Turbo Transfer System (Bio-Rad). The membranes were blocked for 1 hour in 5% non-fat dry milk and then incubated with anti-DPEP1 primary antibody (Cell Signaling Technology, 84292 [mouse specific] and 76290 [human specific]) and β-actin (Sigma-Aldrich, A5316) overnight at 4°C at a 1:1000 dilution. After 3 washes with PBS, membranes were incubated for 2 hours at room temperature with secondary antibody (Peroxidase AffiniPure Donkey Anti-Mouse IgG [Jackson ImmunoResearch, 715-035-150] or Mouse TrueBlot ULTRA Anti-Mouse Ig HRP [Rockland, 18-8817-30] and Anti-Rabbit IgG [whole molecule]–peroxidase antibody produced in goat [Sigma-Aldrich, A6154] or Rabbit TrueBlot Anti-Rabbit IgG HRP [Rockland, 18-8816-31]) before developing with Clarity Western ECL Substrate (Bio-Rad, 170-5061) and autoradiography film with a film processor according to the manufacturer’s protocol or an Amersham Imager 680 (GE Healthcare) according to the manufacturer’s protocol.

### Complete blood counts.

At sacrifice, 500–1000 μL of blood was collected on ice following aortic transection into lavender-top EDTA-coated tubes and run on a ForCyte (Oxford Science Inc.) hematology analyzer by the VUMC TPSR.

### Cell isolation for scRNA-seq.

Mouse tumors were incubated rotating at 4°C for 1.25 hours in a chelating buffer of Dulbecco’s PBS (DPBS) with 20 mM HEPES, 3 mM EDTA, and 0.5 mM DTT, and then gently washed in DPBS. Tumors were then resuspended in DPBS with 5 mg/mL cold active protease and 2.5 mg/ml DNase and incubated at 4°C for 25 minutes with rotation. Tissues were then pipetted 10–20 times with a pipette to liberate single cells, which were passed through a 70 μm filter into a new tube for washes. Cells were washed in DPBS with 0.02% BSA before proceeding immediately to scRNA-seq.

### scRNA-seq.

scRNA-seq was performed using PIPseq T2 V4PLUS kits from Fluent Biosciences. In summary, cells were co-encapsulated with capture beads through vortexing. The resulting emulsion was then incubated to allow for lysis of cells and hybridization of mRNA to barcoded oligos on the beads. Beads were released from droplets and washed, before undergoing reverse transcription and PCR reactions, resulting in amplified barcoded transcript libraries. Libraries were then fragmented, A-tailed, and indexed for sequencing. Sequencing was performed on a NovaSeq XP, targeting 100M reads per sample. scRNA-seq was performed on tumors isolated from 2 WT mice and 3 DPEP1-KO mice.

### scRNA-seq processing.

PIPseeker (Fluent Biosciences) was used to preprocess scRNA-seq reads and to align reads to the reference genome mm10 to generate count matrices of each gene in each cell. Cells with low proportion of uniquely mapping reads (unique molecular identifier [UMI] < 1000), low proportion of expressed genes (<300), or high proportion of reads mapped to the mitochondrial RNA (>20%) were considered as low quality and excluded from downstream analysis. After quality control, scRNA-seq data were normalized using UMI-filtered counts. Common and rare cell populations were identified simultaneously by adaptive *k*-nearest neighbor graph with optimization (78) and visualized by UMAP using Seurat based on the first 30 principal components generated from the top 2000 highly variable genes (79, 80). Cell subpopulations were automatically annotated by single-cell multiresolution marker-based annotation (scMRMA) (81), and then further manually checked by known marker genes. Cell cycle scores of cancer cells were estimated by the CellCycleScoring function in Seurat. Differentially expressed genes between DPEP1-KO and WT in cancer cells were identified by the FindMarkers function in Seurat. Functional class scoring GSEA analysis was performed and plotted with the Genekitr (82) R package (https://github.com/GangLiLab/genekitr).

### Neutrophil binding assay.

Neutrophils were isolated from the peripheral blood of healthy human donors using red blood cell (RBC) lysis followed by MojoSort Whole Blood Human Neutrophil Isolation Kit (BioLegend, 480152) according to the manufacturer’s protocol. Neutrophils were routinely approximately 95% pure as is cited by the sorting protocol. Neutrophils were labeled with CFSE (Thermo Fisher Scientific, C1157) according to the manufacturer’s protocol and diluted to a concentration of 1 × 10^5^ cells/mL, similar to a previously published adhesion assay (16). Neutrophils were mixed with LSALT or a scrambled peptide (Genscript) at a variety of concentrations as shown in the Results and were added as a 500 μL volume onto a confluent monolayer of SW480 or SW620 cells in a 24-well plate. Plates were incubated at 37°C for 30 minutes before 3 PBS washes and fixing in 4% paraformaldehyde. Four fields of view at ×4 or ×10 magnification, depending on the assay, were captured per well, and the number of labeled neutrophils was counted per well by adding all fields of view (FOVs) or considering each FOV as an individual point, depending on the assay. At a minimum, 3 wells per condition were assessed. Binding assays were conducted in triplicate and representative results are included. Viability of cells was not impacted by treatment with LSALT or scrambled peptide at the highest concentration used, as determined by trypan blue staining (Gibco).

### Statistics.

Data involving statistical analysis were plotted using GraphPad Prism 10. Appropriate statistical analysis was conducted by utilization of a 2-tailed Student’s *t* test with Welch’s correction when appropriate or 1-way ANOVA and accounting for non-parametric data when appropriate by Kruskal-Wallis, Dunn’s multiple-comparison, and Mann-Whitney tests. Error bars indicate SEM, unless otherwise denoted. A *P* value of less than 0.05 was considered significant.

### Study approval.

All animal experiments were performed under protocols approved by the Vanderbilt University Animal Care and Use Committee and in accordance with the NIH *Guide for the Care and Use of Laboratory Animals* (National Academies Press, 2011). The experiments on FFPE human colonic tissues and TMAs were approved by umbrella spore IRB no. 070166, TCPS IRB no. 020603, and NA_0080279 (collaborators at John Hopkins University). Blood samples were collected under an IRB-approved protocol (no. 161529) and written informed consent was received prior to participation.

### Data availability.

Curated datasets detailed within this manuscript can be obtained at the discretion of the corresponding author via email inquiry. scRNA-seq data generated in this study have been deposited in the NCBI Gene Expression Omnibus (GEO) database under accession number GSE290572. Values for figures are provided as a Supporting Data Values file.

## Author contributions

SEG conceived the study, designed the experimental methodology, performed experiments, analyzed, interpreted, and visualized the data, and wrote the manuscript. MEB, ZC, RA, PZ, STE, EHG, EGF, RTS, CKS, MJI, FR, CNH, AJS, YX, YW, NOM, and QL performed experiments and analyzed data. MKW and AW analyzed pathology data. GDA analyzed data and provided statistical analysis. MJS, KSL, and RJC supervised the research and edited the manuscript.

## Supplementary Material

Supplemental data

Unedited blot and gel images

Supporting data values

## Figures and Tables

**Table 1:**
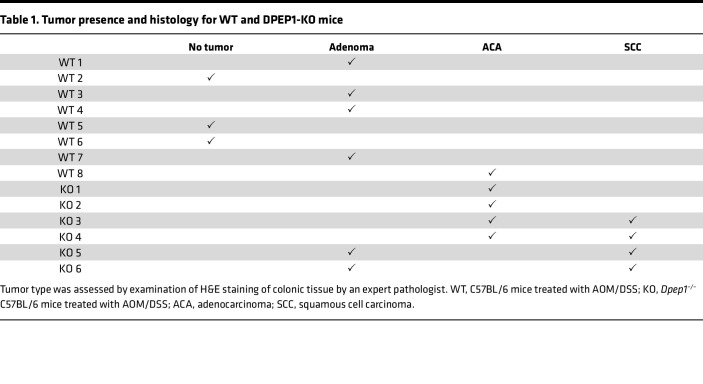
Tumor presence and histology for WT and DPEP1-KO mice

## Version 1 04/03/2025

In-Press Preview

## Version 2 05/08/2025

Electronic publication
