# Supplementary material for: Dipeptidase-1–knockout mice develop invasive tumors with features of microsatellite-unstable colorectal cancer
Source: JCI Insight. 2025 Apr 3;10(9):e186938. doi: 10.1172/jci.insight.186938 (PMC12128987; doi:10.1172/jci.insight.186938)
Supplement: Supplemental data [file jciinsight-10-186938-s106.pdf]

Supplemental Figure 1

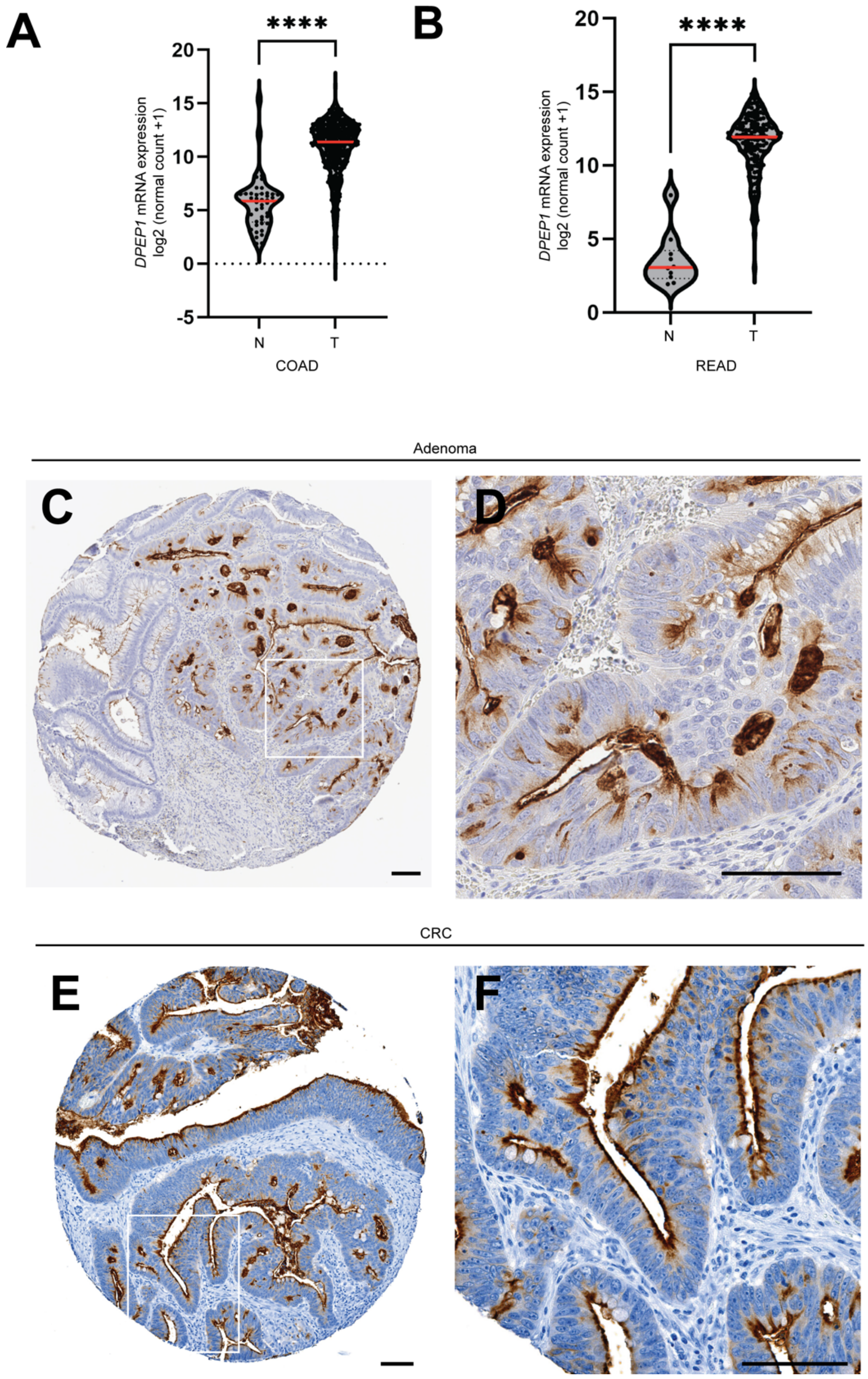

Supplemental Figure 1 legend

DPEP1 is increased in colorectal adenomas and cancers.

DPEP1 mRNA expression in **(A)** colon cancer (n = 492) and **(B)** rectal cancer (n = 170) in comparison to normal adjacent tissues using TCGA datasets. Immunohistochemical staining of DPEP1 in **(C-D)** adenoma (n = 336 total cores assessed) and **(E-F)** CRC tissue samples (n = 249 total cores assessed) with boxed portions magnified to the right. Median denoted in red. N, normal adjacent tissue. T, tumor tissue. Four asterisks denote a p-value < .0001. Scale bar, 100 μm. Data are representative images. COAD, colon adenocarcinoma; READ, rectum adenocarcinoma. Wilcoxon-Mann-Whitney test was used for assessing statistical significance.

Supplemental Figure 2

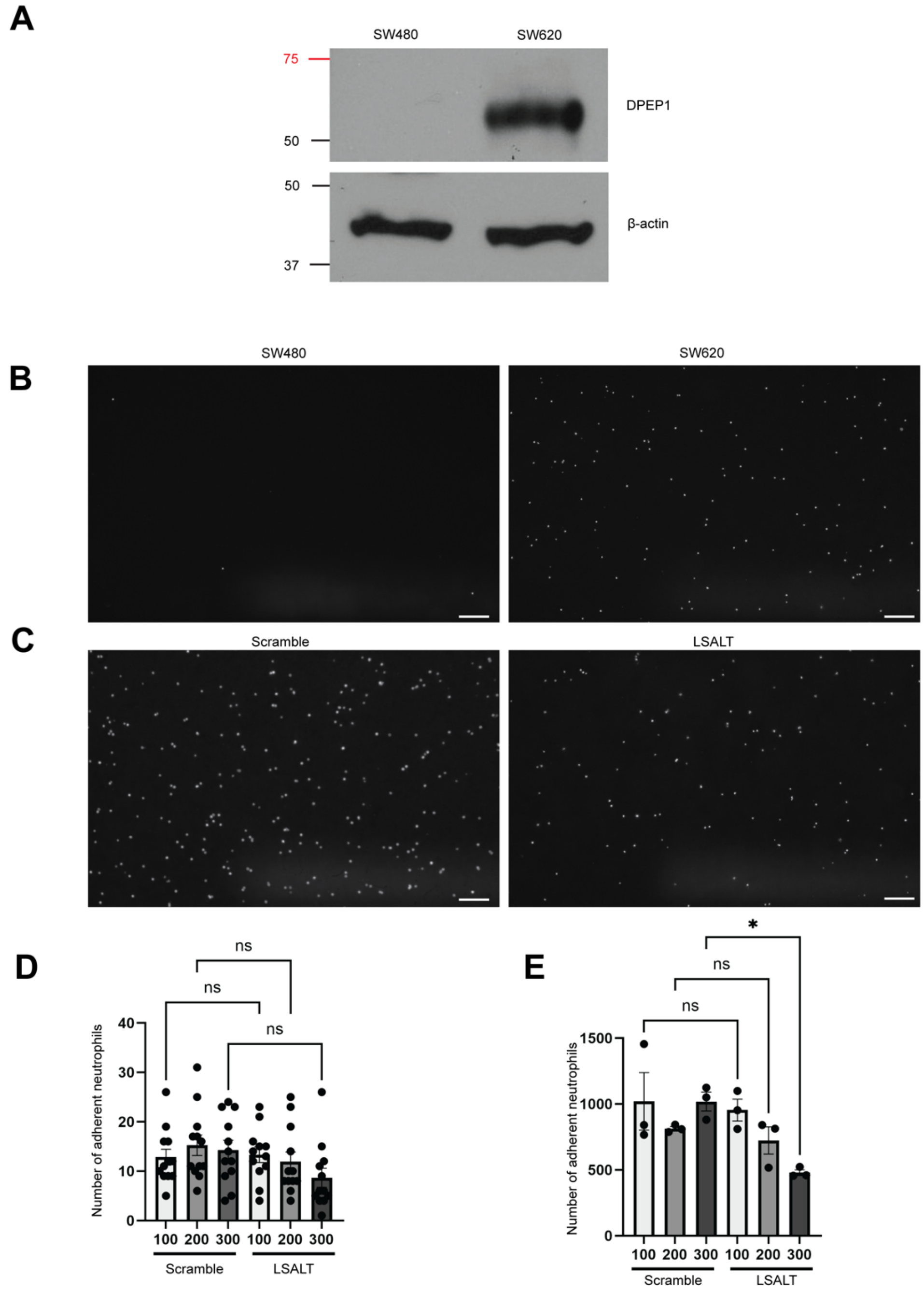

Supplemental Figure 2 Legend

DPEP1 expression in CRC lines is correlated with neutrophil binding ability.

(A) Immunoblot probing DPEP1 in SW480 and SW620 lysates with β-actin used as a loading control **(B)** Representative images of neutrophils bound to SW480 or SW620 cells **(C)** Representative images of neutrophils bound to SW620 cells treated with either 300 μM scrambled or LSALT peptide **(D)** Quantification of neutrophil binding assay for SW480 cells treated with scrambled or LSALT peptide in the designated concentrations in μM as indicated on the plot where each FOV is an individual data point (n = 12 FOV per condition). **(E)** Quantification of neutrophil binding assay for SW620 cells treated with scrambled or LSALT peptide in the designated concentrations in μM as indicated on the plot where each well is an individual data point (n = 3 wells per condition). Data are representative images. Scale bar, 200 μm. Binding assays were conducted in triplicate. Error bars represent SEM. ns, no significance One asterisk denotes a p-value < .05. Kruskal-Wallis test was used for assessing statistical significance.

Supplemental Figure 3

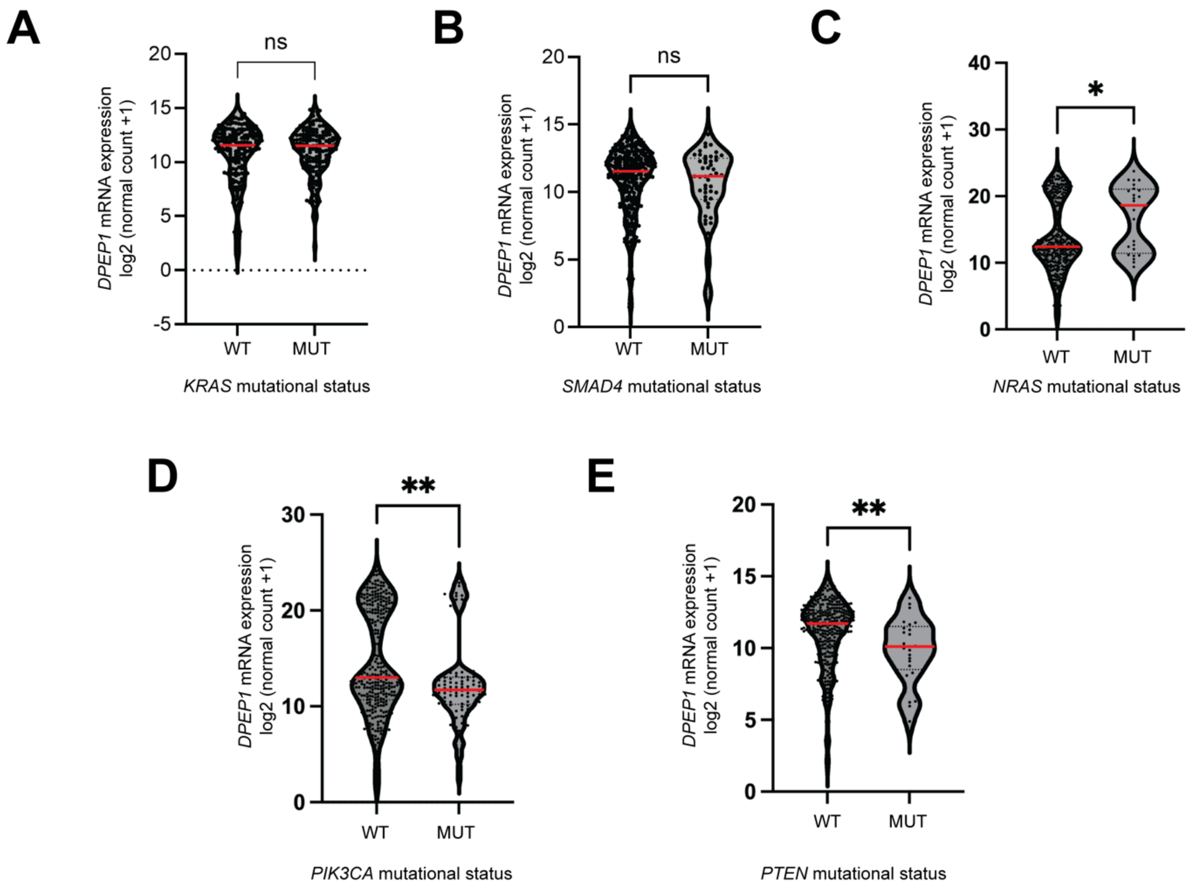

Supplemental Figure 3 Legend

DPEP1 expression is not associated with *KRAS* and *SMAD4* mutations.

DPEP1 mRNA expression of TCGA database COAD and READ cohorts as it relates to **(A)** *KRAS* (n = 355) and **(B)** *SMAD4* (n = 357) mutational status and pathogenic variant mutational status of **(C)** *NRAS* (n = 400), **(D)** *PIK3CA* (n = 397), and **(E)** *PTEN* (n = 352). ns, no significance. One asterisk denotes a p-value < .05. Two asterisks denote a p-value < .01. Wilcoxon-Mann-Whitney test was used for assessing statistical significance**.**

Supplemental Figure 4

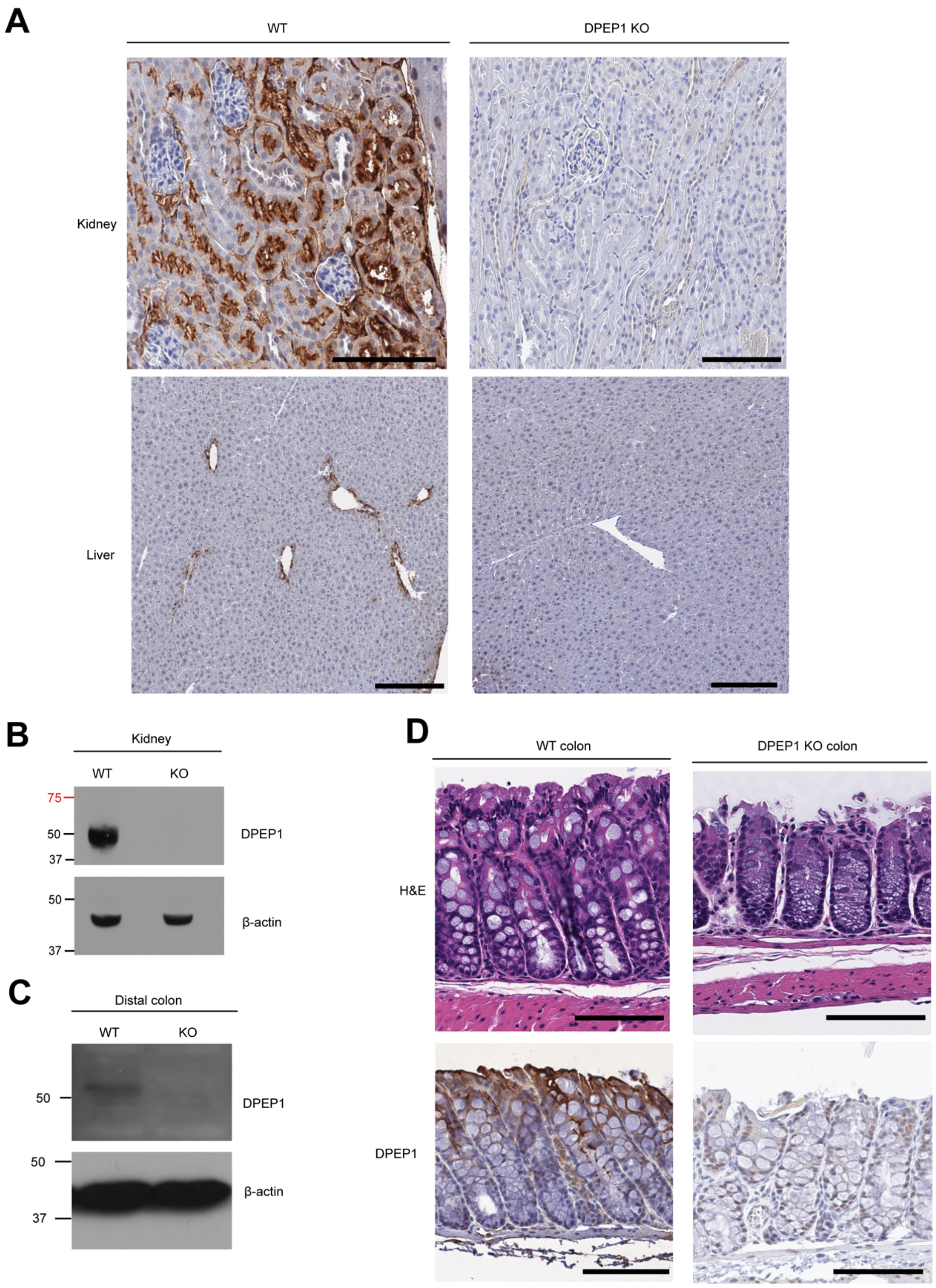

Supplemental Figure 4 Legend

DPEP1 KO is confirmed by IHC and immunoblotting.

(A) DPEP1 IHC for mouse kidney and liver tissues from WT (IHC score = 3) and DPEP1 KO (IHC score = 0) mice. Immunoblot of whole tissue lysate of mouse **(B)** kidney and **(C)** distal colon isolated from WT and DPEP1 KO mice for DPEP1 and β-actin (loading control). **(D)** H&E and DPEP1 IHC for mouse colon tissues from WT (IHC score = 3) and DPEP1 KO (IHC score = 0) mice. Scale bar, 200 μm for liver, 100 μm for all others.

Supplemental Figure 5

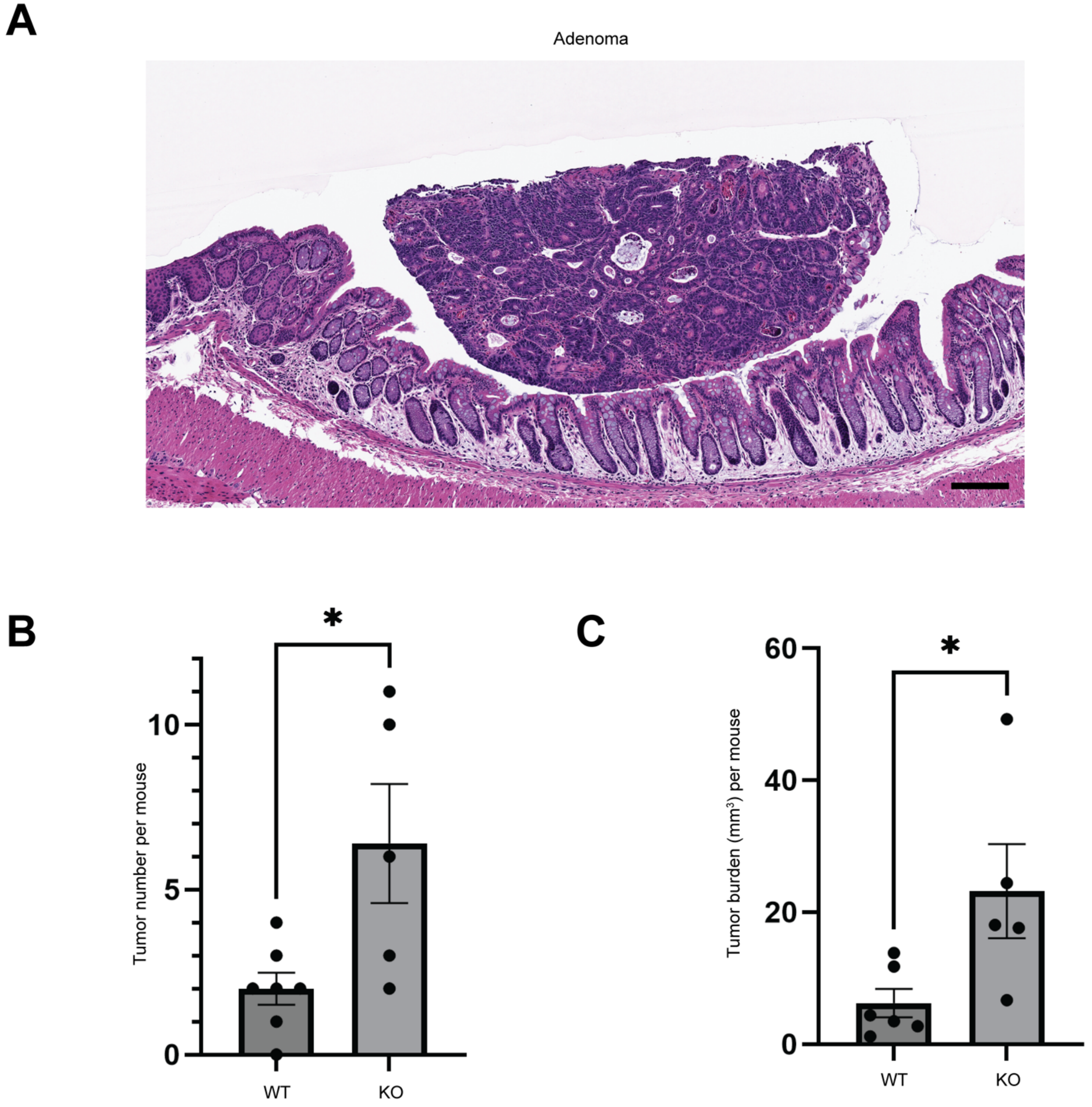

Supplemental Figure 5 Legend

The presence of SCC in DPEP1 KO mice does not skew the observed difference in tumor number and burden between WT and DPEP1 KO mice.

(A) Representative H&E image of WT adenoma from **Figure 3C** at a higher power. Scale bar, 150 μm. **(B)** Quantification of tumor number per mouse comparing WT (n = 7) and DPEP1 KO (n = 5) mice following a regimen of AOM/DSS, only accounting for Ads and ACAs without the inclusion of SCC tumors. **(C)** Quantification of total tumor volume per mouse in WT (n = 7) and DPEP1 KO (n = 5) mice, only accounting for Ads and ACAs without the inclusion of SCC tumors. Error bars represent SEM. One asterisk denotes a p-value $< .05$. Wilcoxon-Mann-Whitney test was used for assessing statistical significance.

Supplemental Figure 6

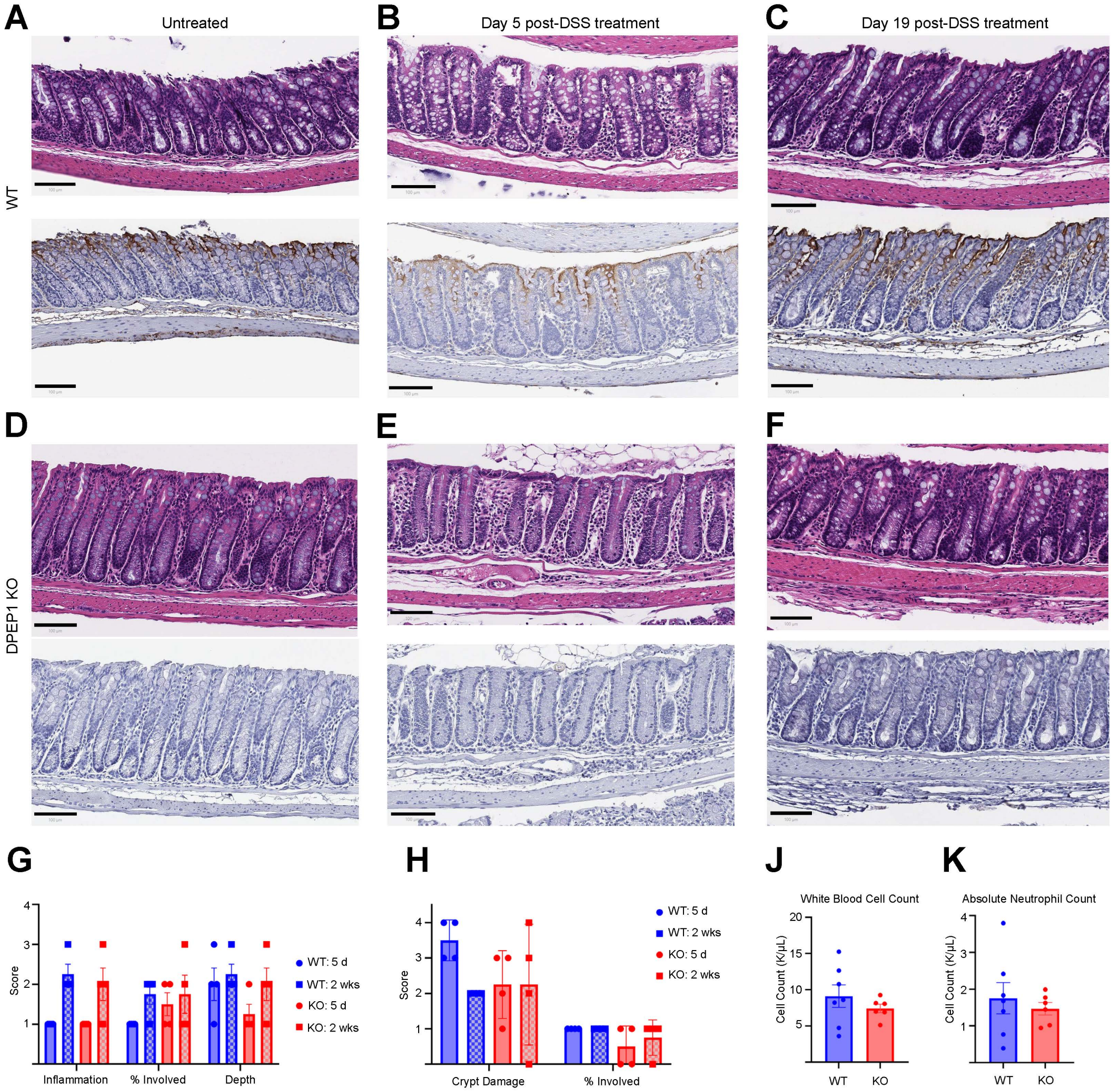

Supplemental Figure 6 Legend

There is no difference in DSS-induced inflammatory response comparing WT and DPEP1 KO mice.

Representative H&E and IHC for DPEP1 in colon tissue for **(A)** WT untreated mice, **(B)** WT mice after 5 days of DSS treatment, or (**C**) WT mice after 2 weeks of resolution post-DSS regimen (19 days post start of DSS treatment). Representative H&E and IHC for DPEP1 in colon tissue for **(D)** DPEP1 KO untreated mice, **(E)** DPEP1 KO mice after 5 days of DSS treatment (day 0 post-DSS regimen), or **(F)** DPEP1 KO mice after 2 weeks of resolution post-DSS regimen (day 14). Expert pathological review of **(G)** DSS-induced inflammation and **(H)** crypt damage after 5 days of DSS treatment and 2 weeks of resolution post-DSS regimen. wks, weeks. N = 4 mice per group. Statistical significance was only observed between 5 d and 2 wks within the same genotype for inflammation scores. **(J)** White blood cell count and **(K)** absolute neutrophil count from peripheral blood of tumor-bearing mice. Statistical significance was not observed for **H** - **K** comparing WT (n = 7) and DPEP1 KO (n = 6) mice. Scale bar, 100 µm. Error bars represent SEM. Kruskal-Wallis and Wilcoxon-Mann-Whitney test were used where appropriate for assessing statistical significance.

Supplemental Figure 7

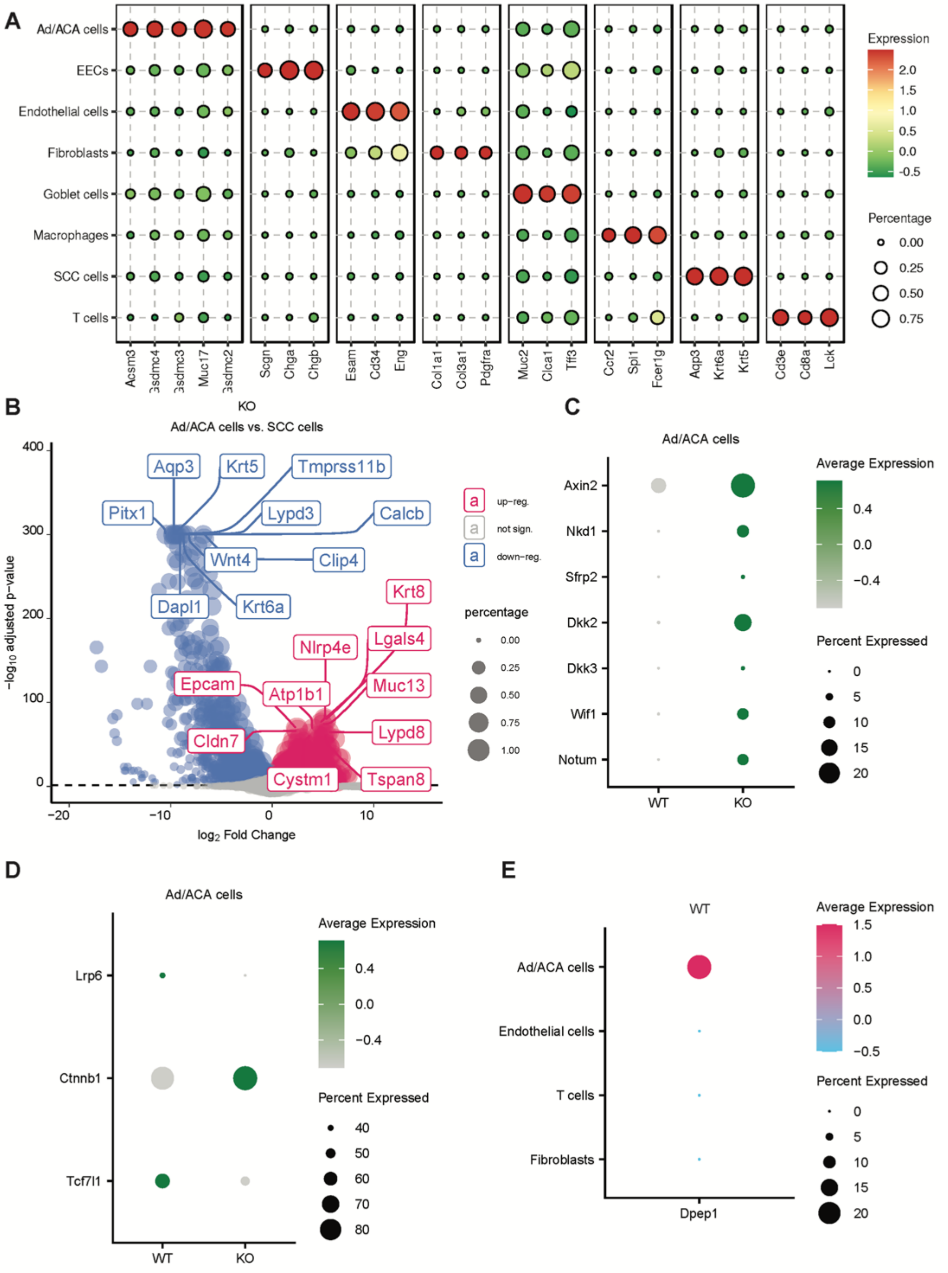

Supplemental Figure 7 Legend

A lack of DPEP1 results in two tumor phenotypes and impacts Wnt signaling mediators.

(A) Relative gene expression of representing cell type markers shown in bubble heatmap plot. **(B)** Volcano plot showing the DE genes between Ad/ACA cells and SCC in DPEP1 KO group; Pink dots shows the up-regulated genes in Ad/ACA cells, while blue dots shows the down-regulated genes in Ad/ACA cells; Size of dots show the larger percentage of cells expressing the gene in either groups; maximum y-axis was set to 300 for genes with adjusted p-value equal to 0; **(C)** Dot plot of Wnt negative regulator significantly DE genes between DPEP1 KO and WT groups in Ad/ACA cells (*Notom* Padj = 4.5e-32; *Wif1* Padj = 8.7e-31; *Dkk3* Padj = 0.014; *Dkk2* Padj = 6.6e-53; *Sfrp2* Padj = 7.1e-3; *Nkd1* Padj = 1.1e-34; *Axin2* Padj = 3.1e-13); **(D)** Dot plot of Wnt activator significantly DE genes between DPEP1 KO and WT groups in Ad/ACA cells (*Tcf7l1* Padj = 7.0e-18; *Ctnnb1* Padj = 1.2e-20; *Lrp6* Padj = 5.1e-5). **(E)** Dot plot of *Dpep1* expression in WT Ad/ACA cells, Endothelial cells, T cells, and Fibroblasts. Padj, adjusted p-value.

Supplemental Figure 8

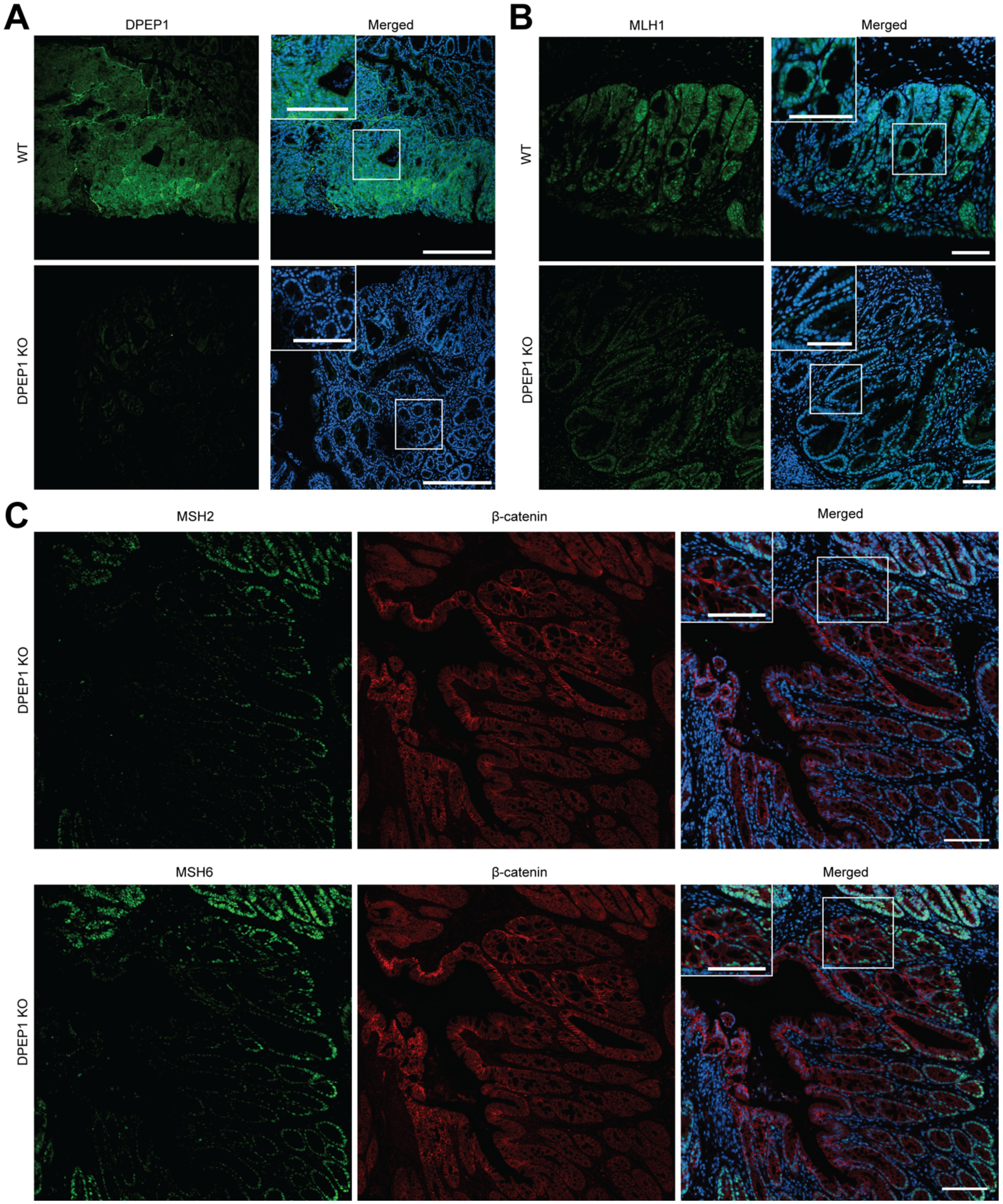

Supplemental Figure 8 Legend

DPEP1 KO is confirmed by immunofluorescence and is associated with reduced MLH1.

Representative immunofluorescence images showing **(A)** DPEP1, **(B)** MLH1, or **(C)** MSH2, MSH6, and β-catenin staining and merged images with DAPI for WT Ads and DPEP1 KO ACAs formed by AOM/DSS. Scale bar, 500 μm, insets 250 μm, for **A** and 100 μm for **B** and **C**. Representative images are a result of staining tumors from two cohorts described in the Methods where experiments were done in triplicate from WT (n = 12) and DPEP1 KO (n = 10) tumors.

Supplemental Figure 9

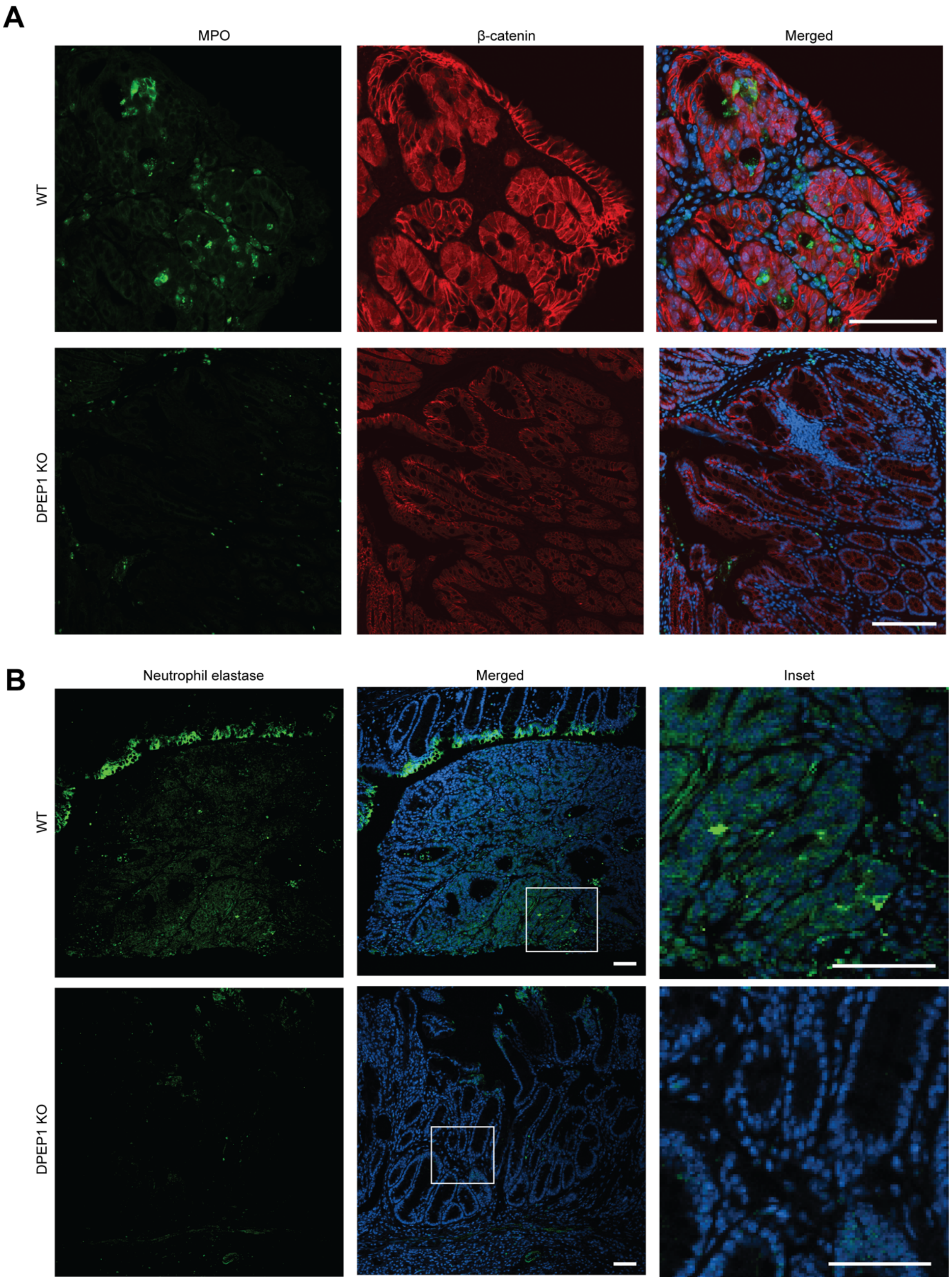

Supplemental Figure 9 Legend

A lack of DPEP1 leads to a decrease in activated neutrophils.

Representative immunofluorescence images showing **(A)** MPO and β-catenin or **(B)** neutrophil elastase staining and merged images with DAPI for WT Ads and DPEP1 KO ACAs formed by AOM/DSS. Scale bar, 50 μm for **A** and 100 μm for **B**. Representative images are a result of staining tumors from two cohorts described in the Methods where experiments were done in triplicate from WT (n = 12) and DPEP1 KO (n = 10) tumors.
