# Supplementary material for: Dipeptidase-1–knockout mice develop invasive tumors with features of microsatellite-unstable colorectal cancer
Source: JCI Insight. 2025 Apr 3;10(9):e186938. doi: 10.1172/jci.insight.186938 (PMC12128987; doi:10.1172/jci.insight.186938)
Supplement: Unedited blot and gel images [file jciinsight-10-186938-s107.pdf]

Full unedited blot for
Supplemental Figure 2A

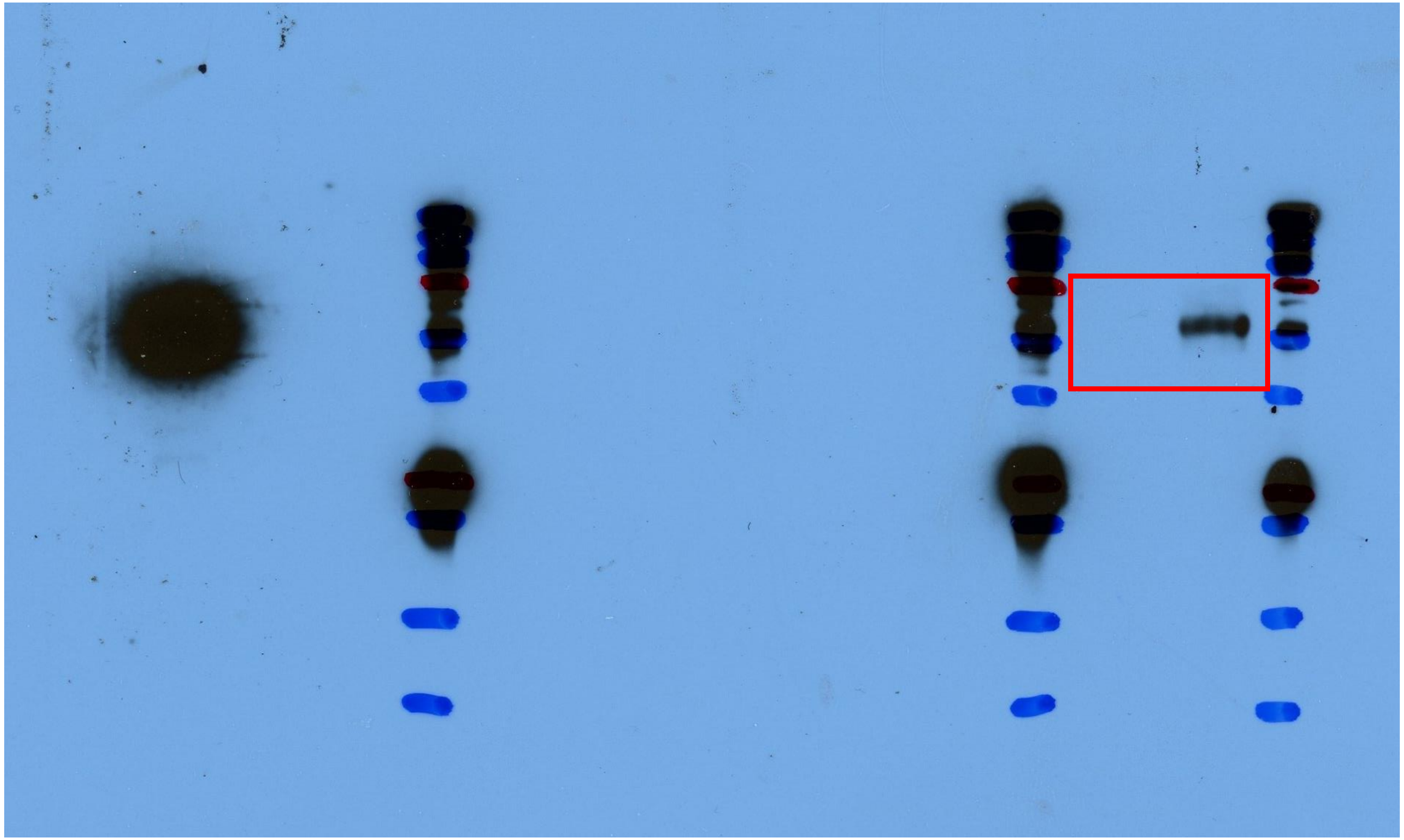

Supplemental Figure 2A
Top (DPEP1)

Full unedited blot for
Supplemental Figure 2A

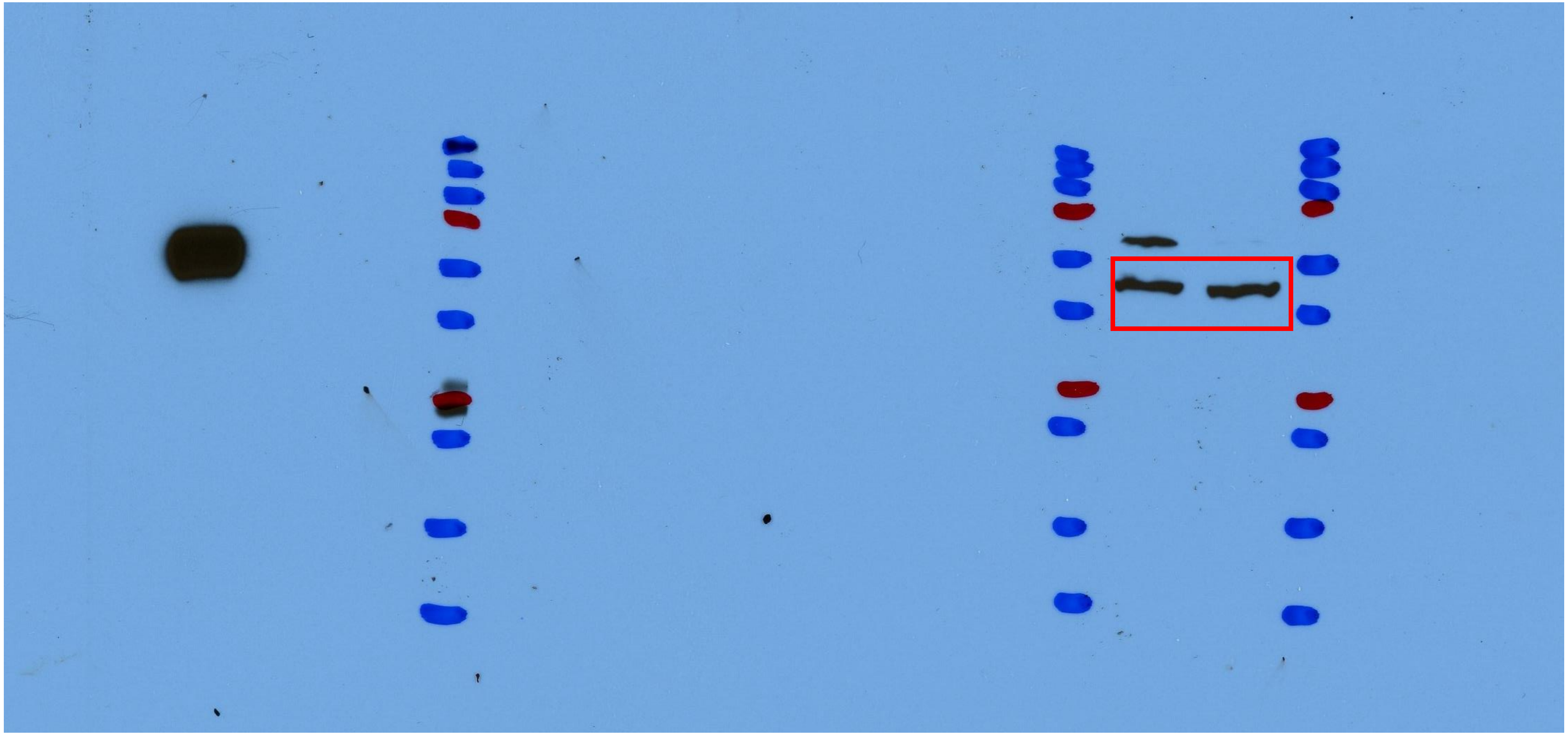

Supplemental Figure 2A
Bottom (β-actin)

Full unedited blot for Supplemental Figure 4B

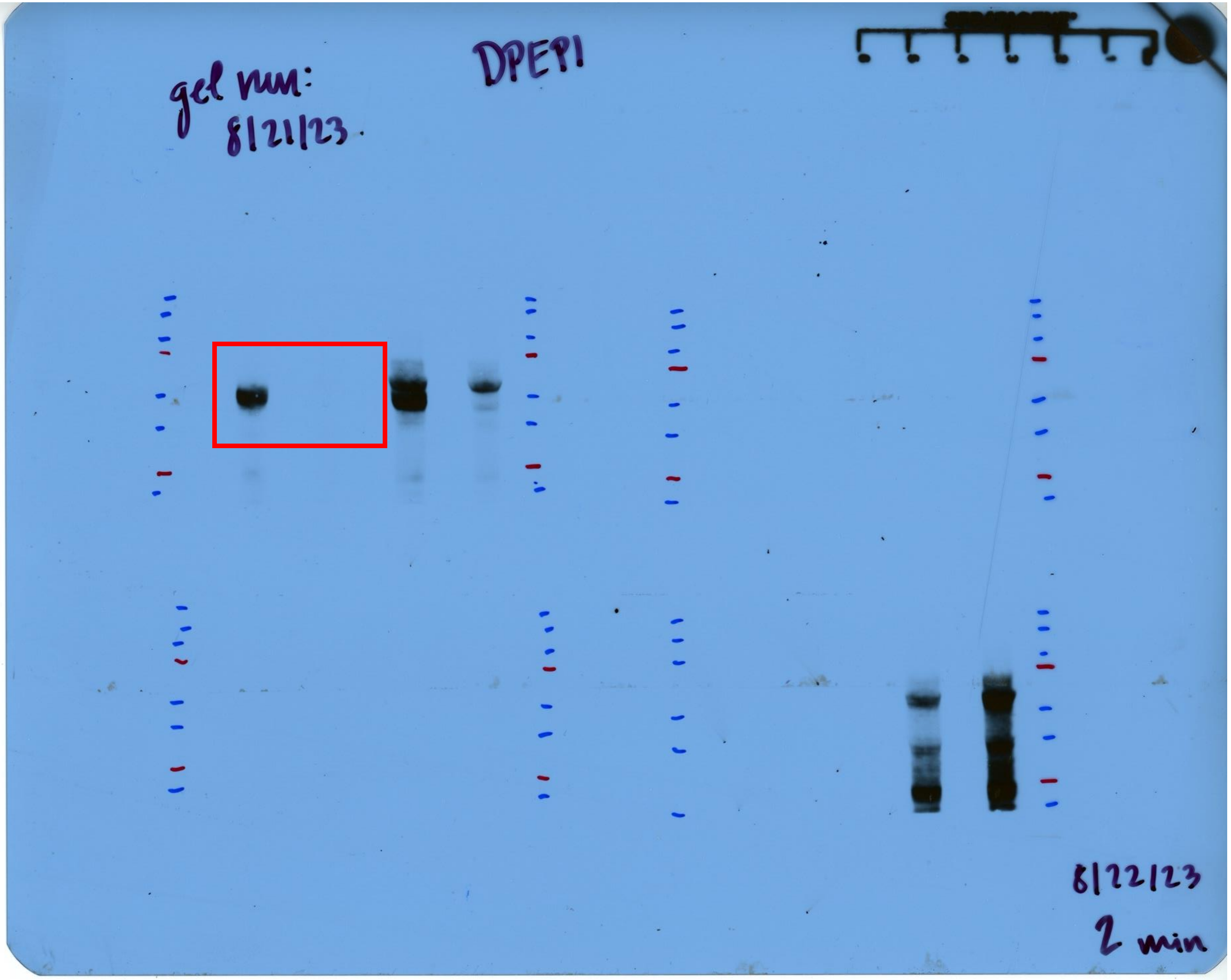

Supplemental Figure 4B Top (DPEP1)

Full unedited blot for Supplemental Figure 4B

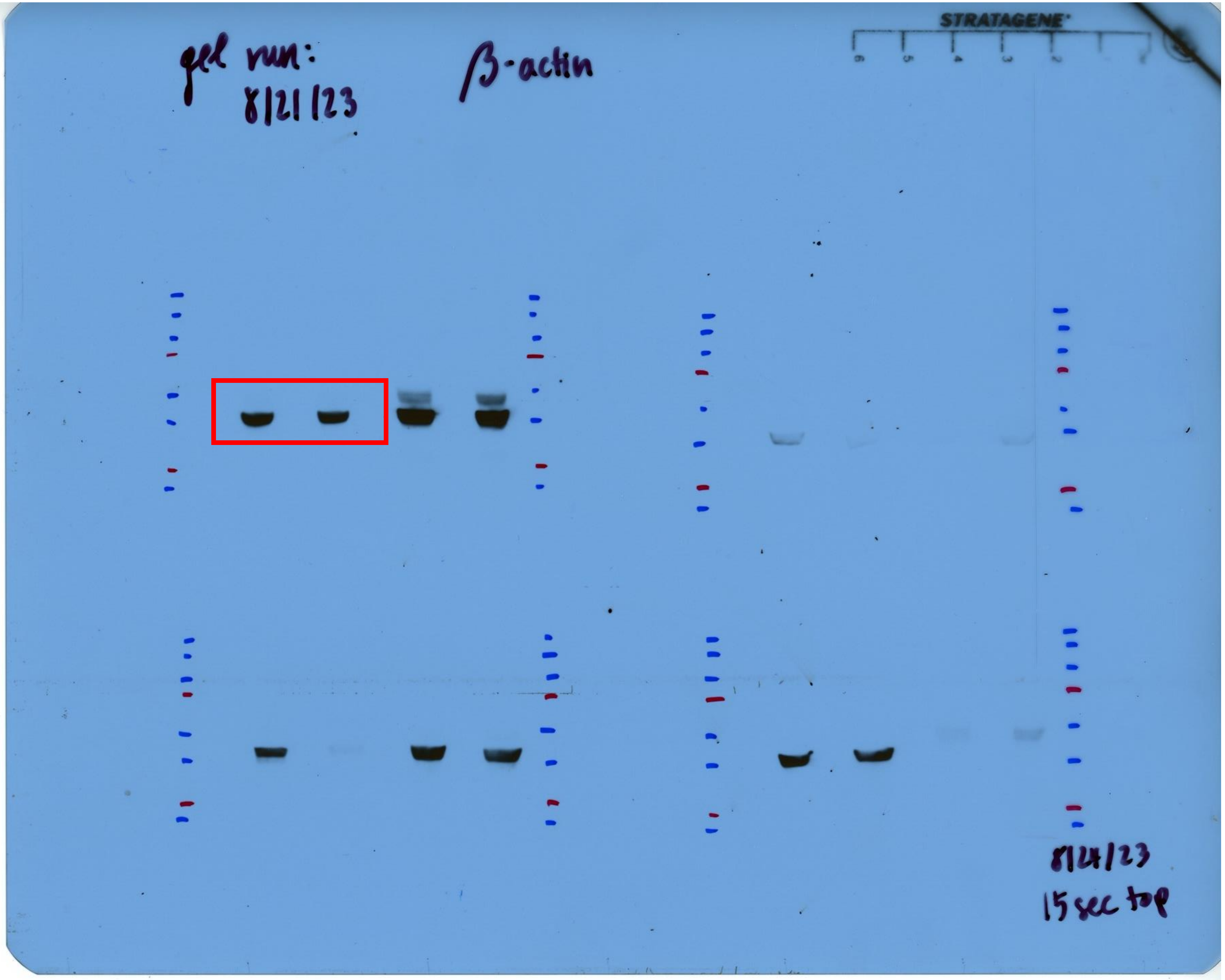

Supplemental Figure 4B Bottom (β-actin)

Full unedited blot for
Supplemental Figure 4C

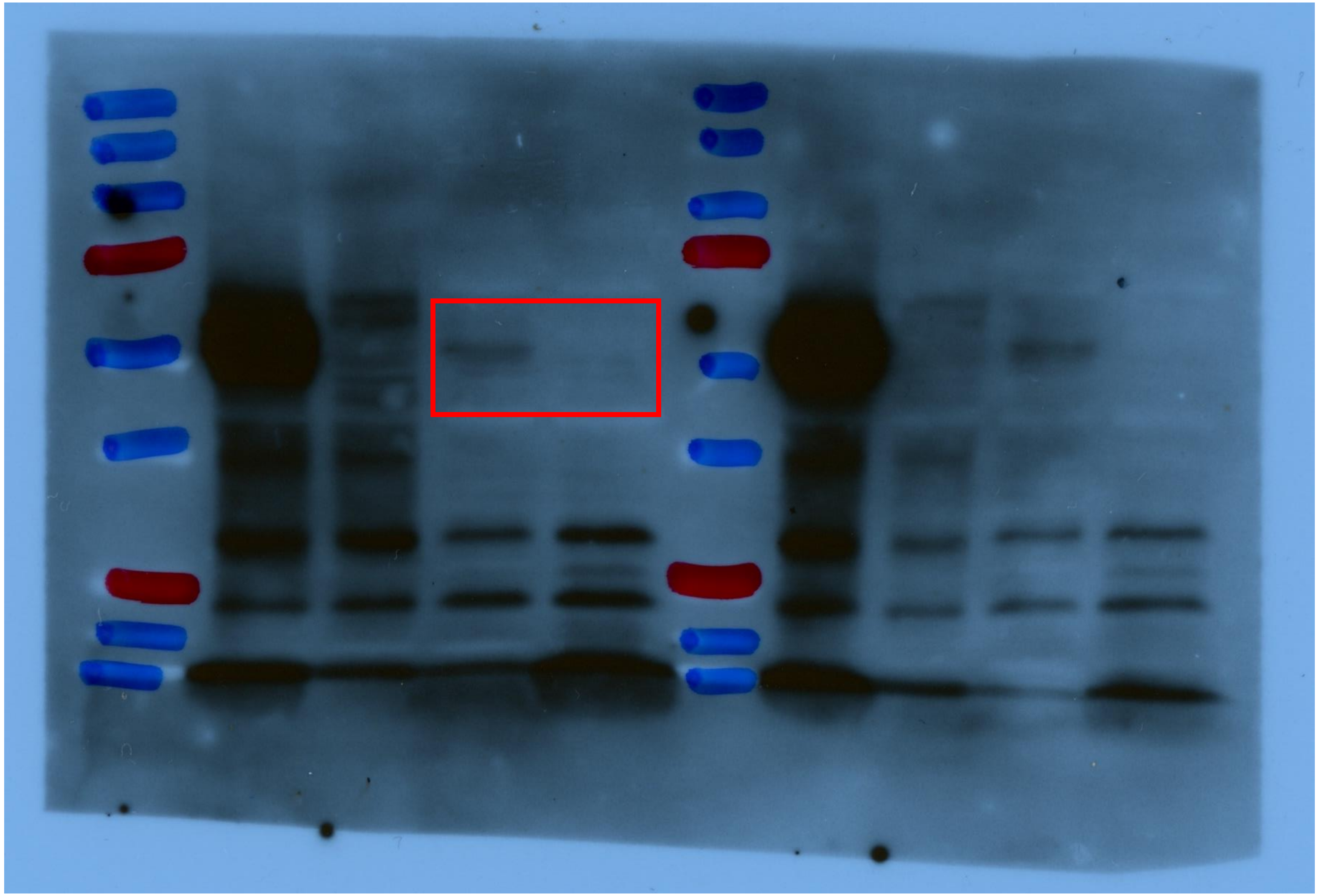

Supplemental Figure 4C
Top (DPEP1)

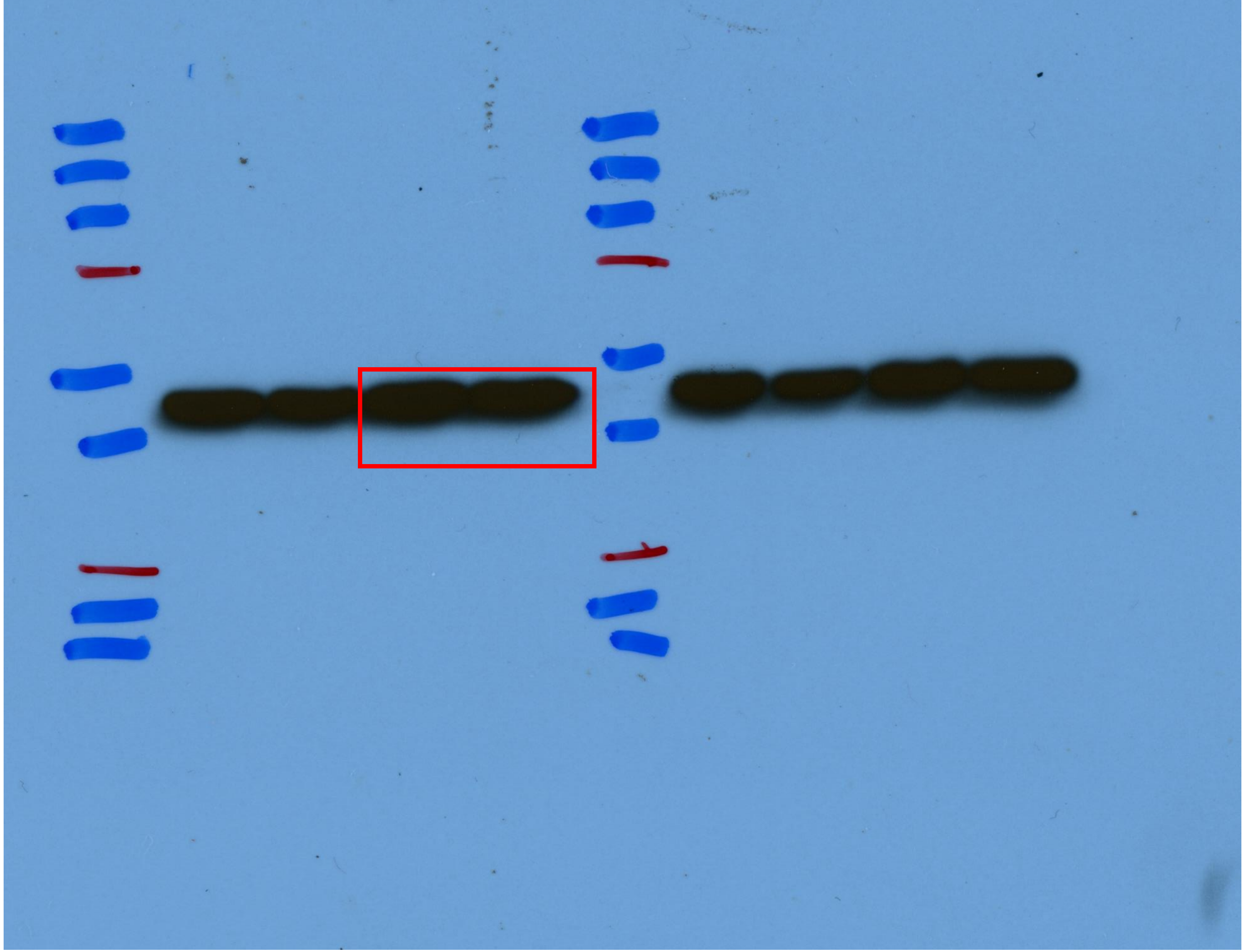

Full unedited blot for
Supplemental Figure 4C

Supplemental Figure 4C
Bottom (β-actin)
